# Temperature Variability and Mortality: A Multi-Country Study

**DOI:** 10.1289/EHP149

**Published:** 2016-06-03

**Authors:** Yuming Guo, Antonio Gasparrini, Ben G. Armstrong, Benjawan Tawatsupa, Aurelio Tobias, Eric Lavigne, Micheline de Sousa Zanotti Stagliorio Coelho, Xiaochuan Pan, Ho Kim, Masahiro Hashizume, Yasushi Honda, Yue Leon Guo, Chang-Fu Wu, Antonella Zanobetti, Joel D. Schwartz, Michelle L. Bell, Ala Overcenco, Kornwipa Punnasiri, Shanshan Li, Linwei Tian, Paulo Saldiva, Gail Williams, Shilu Tong

**Affiliations:** 1 Department of Epidemiology and Biostatistics, School of Public Health, University of Queensland, Brisbane, Australia; 2 Department of Social and Environmental Health Research, London School of Hygiene & Tropical Medicine, London, United Kingdom; 3 Health Impact Assessment Division, Department of Health, Ministry of Public Heath, Thailand; 4 Institute of Environmental Assessment and Water Research, Spanish Council for Scientific Research, Barcelona, Spain; 5 School of Epidemiology, Public Health and Preventive Medicine, University of Ottawa, Ottawa, Canada; 6 Laboratory of Experimental Air Pollution, Department of Pathology, School of Medicine, University of São Paulo, São Paulo, Brazil; 7 Department of Occupational and Environmental Health, School of Public Health, Peking University, Beijing, China; 8 Graduate School of Public Health, Seoul National University, Seoul, Republic of Korea; 9 Department of Pediatric Infectious Diseases, Institute of Tropical Medicine, Nagasaki University, Nagasaki, Japan; 10 Faculty of Health and Sport Sciences, University of Tsukuba, Tsukuba, Japan; 11 Department of Environmental and Occupational Medicine, National Taiwan University, Taipei, Taiwan; 12 Department of Public Health, National Taiwan University, Taipei, Taiwan; 13 Department of Environmental Health, Harvard T.H. Chan School of Public Health, Boston, Massachusetts, USA; 14 School of Forestry and Environmental Studies, Yale University, New Haven, Connecticut, USA; 15 Laboratory of Management in Public Health, Chisinau, Republic of Moldova; 16 Division of Epidemiology and Biostatistics, School of Public Health, University of Hong Kong, Hong Kong, China; 17 School of Public Health and Social Work, and; 18 Institute of Health and Biomedical Innovation, Queensland University of Technology, Brisbane, Australia

## Abstract

**Background:**

The evidence and method are limited for the associations between mortality and temperature variability (TV) within or between days.

**Objectives:**

We developed a novel method to calculate TV and investigated TV-mortality associations using a large multicountry data set.

**Methods:**

We collected daily data for temperature and mortality from 372 locations in 12 countries/regions (Australia, Brazil, Canada, China, Japan, Moldova, South Korea, Spain, Taiwan, Thailand, the United Kingdom, and the United States). We calculated TV from the standard deviation of the minimum and maximum temperatures during the exposure days. Two-stage analyses were used to assess the relationship between TV and mortality. In the first stage, a Poisson regression model allowing over-dispersion was used to estimate the community-specific TV-mortality relationship, after controlling for potential confounders. In the second stage, a meta-analysis was used to pool the effect estimates within each country.

**Results:**

There was a significant association between TV and mortality in all countries, even after controlling for the effects of daily mean temperature. In stratified analyses, TV was still significantly associated with mortality in cold, hot, and moderate seasons. Mortality risks related to TV were higher in hot areas than in cold areas when using short TV exposures (0–1 days), whereas TV-related mortality risks were higher in moderate areas than in cold and hot areas when using longer TV exposures (0–7 days).

**Conclusions:**

The results indicate that more attention should be paid to unstable weather conditions in order to protect health. These findings may have implications for developing public health policies to manage health risks of climate change.

**Citation:**

Guo Y, Gasparrini A, Armstrong BG, Tawatsupa B, Tobias A, Lavigne E, Coelho MS, Pan X, Kim H, Hashizume M, Honda Y, Guo YL, Wu CF, Zanobetti A, Schwartz JD, Bell ML, Overcenco A, Punnasiri K, Li S, Tian L, Saldiva P, Williams G, Tong S. 2016. Temperature variability and mortality: a multi-country study. Environ Health Perspect 124:1554–1559; http://dx.doi.org/10.1289/EHP149

## Introduction

Time series data on daily air pollution concentrations, weather conditions, and daily measures of health outcomes (e.g., mortality, hospital admissions), have been used to assess how environmental factors may contribute to short-term (days to weeks after the environmental exposure) increases in mortality/morbidity (Bhaskaran et al. 2013; Samet et al. 2000). To date, numerous time series analyses have shown that both cold and hot temperatures are associated with increased risks for a number of health outcomes (Basu and Samet 2002; Basu 2009; Ye et al. 2012). These findings have important implications for understanding the health effects of climate change (Field 2012). However, because climate change increases both the average values and the variability of temperature (Stocker 2014), the health impacts of unstable weather remain unclear (Zanobetti et al. 2012). People may adapt to the usual temperature but may not adapt to the variable temperature. Thus, additional evidence is needed for assessing the health impacts of temperature variability (TV) locally, regionally, and globally.

At the present time, two indices, intraday TV (e.g., diurnal temperature range) and interday TV (e.g., temperature change between neighboring days), have been used to assess the associations between short-term unstable weather and population health (Guo et al. 2011b; Lin et al. 2013; Qiu et al. 2013; Yang et al. 2013). In addition, some studies have used the standard deviation of summer daily mean temperature to represent summer long-term TV (Shi et al. 2015; Zanobetti et al. 2012); this is also a type of interday variability. All of the abovementioned studies assessed the relationships between health outcomes and intra- and interday variability separately. However, because unstable weather is a continuous process, impacts on health may be better captured by considering the intraday and interday variability together when assessing the associations between TV and population health.

In addition, most previous studies of unstable weather and health risks were from one city, one region, or one country, and used different methods. These differences make it difficult to compare the findings directly. We have recently established a Multi-Country multi-City (MCC) collaborative network to assess the effects of weather on mortality globally (Gasparrini et al. 2015a, 2015b; Guo et al. 2014). In this study, we developed a novel method to calculate TV that includes both intraday and interday TV, and we examined TV-mortality associations using the MCC data.

## Methods

### Data Collection

**Figure 1:**
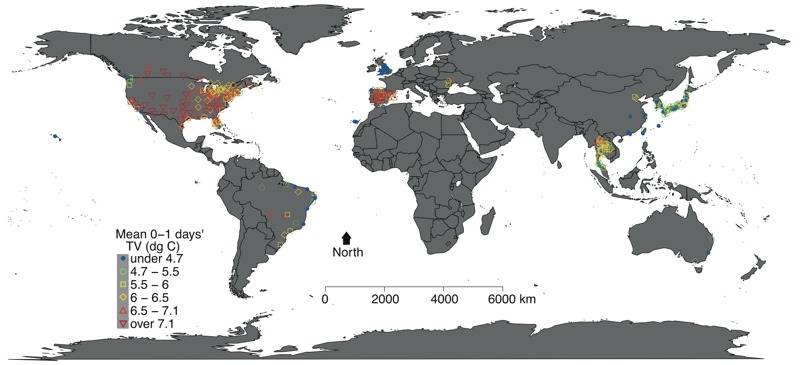
Locations of study areas and their mean values of 0–1 days’ temperature variability (°C). The map is freely downloaded from the “maps” package of R software. TV, Temperature variability

The MCC data set has been described in previous publications (Gasparrini et al. 2015a, 2015b; Guo et al. 2014). In brief, we obtained daily counts of all cause/non-accidental deaths and weather conditions in 372 communities from 12 countries/regions: Australia (3 cities during 1988–2008), Brazil (18 cities during 1997–2011), Canada (26 cities during 1986–2009), China (6 cities during 2002–2007), Japan (47 prefectures during 1972–2012), Moldova (4 cities during 2001–2010), South Korea (7 cities during 1992–2010), Spain (51 cities during 1990–2010), Taiwan (3 cities during 1994–2007), Thailand (62 provinces during 1999–2008), the United Kingdom (10 regions during 1993–2006), and the United States (135 cities during 1985–2006). Daily weather data included the daily minimum, mean, and maximum temperatures and the relative humidity. The locations are displayed in Figure 1. The Supplemental Material provides the details for data collection in “Data Collection,” and Table S1 shows the list of locations.

### Calculation of Temperature Variability

Evidence shows that the associations between both intraday and interday TV and health outcomes last for several days (Lin et al. 2013; Yang et al. 2013), suggesting that the impacts of TV on health should be a continuous process. However, these associations were assessed separately, which makes it difficult to assess the overall effects of TV. In addition, putting intraday and interday TV into the same model might make the model unstable and lead to invalid effect estimates because there might be strong collinearity between intraday and interday TV, particularly when considering lag effects. In this study, we developed a composite index of intraday and interday TV by calculating the standard deviation (SD) of the minimum and maximum temperatures (MinTemp and MaxTemp, respectively) during the exposure days. For example, the TV for the preceding 2 days’ exposure was calculated as follows: TV_0–1_ = SD (MinTemp_lag0_, MaxTemp_lag0_, MinTemp_lag1_, MaxTemp_lag1_). The TV for the preceding 3 days’ exposure was calculated by TV_0–2_ = SD (MinTemp_lag0_, MaxTemp_lag0_, MinTemp_lag1_, MaxTemp_lag1_, MinTemp_lag2_, MaxTemp_lag2_). This method can account for both intraday and interday TV as well as for the lag effects of TV.

### Data Analysis

***Analytic plan.*** The TV-mortality association was investigated with a two-stage analytic approach using time-series data from the 372 communities in the 12 countries/regions. In the first stage, we applied a time series model to each community’s data to estimate the city-specific TV-mortality relationship. These estimated relationships were then pooled in the second stage at the country level with a meta-analysis. This approach has been described previously (Gasparrini et al. 2012; Gasparrini and Armstrong 2013).

***First stage of analysis.*** In the first stage, we used a regression model to obtain community-specific estimates assuming a quasi-Poisson distribution allowing for over-dispersed death counts, which follows a standard analytical approach for time-series data (Bhaskaran et al. 2013). We used a linear function for TV because previous studies have suggested that diurnal temperature range has a linear effect on health, and both large decreases and large increases in temperature between neighboring days increases the risk of health outcomes. Long-term trends and seasonality were controlled for using a natural cubic spline with 7 degrees of freedom per year for time. A categorical variable was used to control for the confounding effect of day of the week. We also controlled for the nonlinear and delayed effects of daily mean temperature using a distributed lag nonlinear model (Gasparrini et al. 2010). A natural cubic spline with 4 degrees of freedom was used for the daily mean temperature, and a natural cubic spline with 4 degrees of freedom was used to capture the lags over time up to 21 days. We placed three internal knots at equally spaced temperature percentiles (25th, 50th, and 75th) and two internal knots at equally spaced log-values of lag (approximately 1.4 and 5.5 days), plus intercept. The choice of 21 days for the lag period was motivated by previous studies showing that effects of cold temperature were more delayed and spread over the previous weeks of exposure, whereas the effects of hot temperatures were more acute and were based on same-day and the previous few days’ exposures (Gasparrini and Armstrong 2013; Guo et al. 2014). We controlled for daily mean temperature rather than for daily minimum and maximum temperatures because daily mean temperature represents the exposure throughout the whole day and night and corresponds to the daily count of deaths.

We assessed several lengths of exposure to TV separately, for example, the preceding 2 days (same day and 1 day before, 0–1 days), the preceding 3 days (same day, 1 and 2 days before, 0–2 days), up to the preceding 8 days (0–7 days), to understand which length of TV exposure was associated with mortality risks.

We calculated the community-specific effect estimates of death associated with 1 interquantile range (IQR; for each community) increase of TV because most communities had nonoverlapping ranges of TV (see Table S1). In addition, a sensitivity analysis looking at the effect per 1°C increase in TV showed that effect is more heterogeneous than the effect per IQR change in TV in the meta-analysis. The values of IQR for each community are shown in Table S1.

To examine whether the effects of TV on mortality differed by different seasonal characteristics, we conducted stratified analyses for the cold season (4 coldest months), the hot season (4 hottest months), and the moderate season (remainder of the year), using an interactive term between TV and seasons (as a categorical variable) in the community-specific regression model. We defined the cold season as the 4 months with the lowest monthly mean temperature for each community, the hot season as the 4 months with the highest monthly mean temperature, and the moderate season as the 4 months not included in the hot and cold seasons.

***Second stage of analysis.*** In the second stage, a meta-analysis was used to pool the community-specific effect estimates obtained from the first-stage model. The meta-analyses were fitted using a random effects model by maximum likelihood and were applied in each country to obtain national pooled estimates.

Studies have reported that people may have the ability to adapt to their local climate. In order to understand whether the associations between TV and mortality are different by climate (for example, whether warm/cold locations have higher effect estimates for TV-mortality associations than cold/warm locations), we divided 372 communities into four groups (cold, moderate cold, moderate hot, and hot areas) by the quantiles (≤ 25th, 25th–50th, 50th–75th, and > 75th) of their annual mean temperatures during the study period (each community had one value for annual mean temperature) (see Figure S1). Meta-analyses were used to pool the community-specific effect estimates obtained from the first-stage model for these four groups.

The TV-mortality associations were expressed as the percent increase [95% confidence interval (CI)] in mortality associated with an increase in IQR (for each community) of TV.

Sensitivity analyses were performed on the parameters for the community-specific model to test the robustness of our results. We changed lag days to 28 days to examine whether using 21 lag days was sufficient to control for the temperature effects on mortality. We modified the degrees of freedom (df) for temperature (3–6 df). We included relative humidity into the analyses. We also included heat waves and cold spells into the analyses because they might be responsible for increases in TV. Heat waves were defined as temperatures > 95th percentile of the daily mean temperature for that community with duration > 2 days, and cold spells were defined as temperatures < 5th percentile of the daily mean temperature with duration > 2 days. We also controlled for daily minimum and daily maximum temperatures instead of the daily mean temperature, using the same distributed lag nonlinear model, to check whether daily minimum and maximum temperatures confounded the associations between TV and mortality.

In addition, an approach known as generalization of Granger causality (Flanders et al. 2011) was used to check the possibility of residual confounding and potential cause–effect association between TV and mortality. Briefly, we examined the associations between daily mortality and future 1–7 days’ TV. If this future exposure was associated with mortality, it was an indication of residual confounding with the model. If there was no association between this future exposure and mortality, there was no residual confounding, and there was a cause–effect relationship between TV and mortality.

R software (v.3.0.1, R Project for Statistical Computing) was used for data analysis. The “dlnm” package was used to create the distributed lag nonlinear model (Gasparrini et al. 2010), and the “mvmeta” package was used to fit the meta-analyses (Gasparrini et al. 2012).

This study was approved by the Behavioural & Social Sciences Ethical Review Committee, University of Queensland.

## Results

This study included 372 communities and covered the period from 1972 to 2012, with different years of data for different regions (Table 1). The total death counts were > 83 million. Thailand had the hottest climate pattern, and Canada had the coldest. Table S1 shows that the daily average counts of death, average temperatures, and TV varied greatly by community.

**Table 1:** Summary of the study periods, number of deaths, and mean temperatures in the 12 countries/regions.

Country/region	Period	Number of communities	Number of deaths	Mean temperature (°C)
Australia	1988–2009	3	1,184,154	18.1
Brazil	1997–2011	18	3,435,535	24.2
Canada	1986–2009	26	2,989,901	6.8
China	2002–2007	6	558,959	18.4
Japan	1972–2012	47	33,511,400	15.1
Korea	1992–2010	7	1,511,996	13.7
Moldova	2001–2010	4	59,906	10.7
Spain	1990–2010	51	3,480,531	15.5
Taiwan	1994–2007	3	688,394	24.0
Thailand	1999–2008	62	1,827,853	27.6
United Kingdom	1990–2012	10	11,636,089	10.3
United States	1985–2006	135	22,896,409	14.9

**Figure 2:**
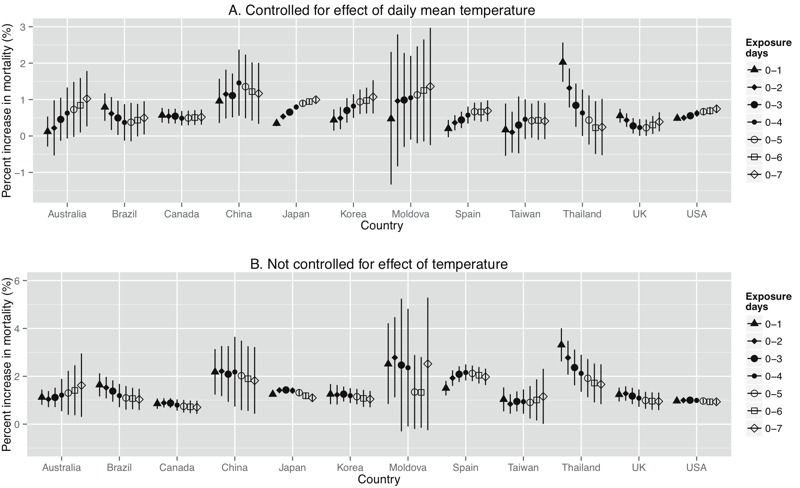
Percent change (95% confidence interval) in mortality associated with an interquantile (for each community) increase in temperature variability (°C) on different exposure days, (*A*) after controlling for the effect of daily mean temperature, (*B*) without controlling for the effect of temperature.

It is clear that the associations between TV (associated with one IQR increase) and mortality with adjustment for the effects of daily mean temperature (Figure 2A) were lower than those without adjustment for the effects of temperature (Figure 2B) during all exposure days in all countries/regions. In addition, the highest effect estimates appeared on different exposure days in different countries. For the models controlling for the effects of daily mean temperature, the highest effect estimates appeared at 0–7 days of exposure for Australia, Japan, Korea, Spain, and the United States; 0–1 days for Brazil, Thailand, and the United Kingdom; and 0–4 days for China. Canada had the same effect estimates on all types of exposure days. The effect estimates varied by community (results not shown).

In general, there were positive associations between TV and mortality in all seasons in all countries/regions (Table 2). The effect estimates for TV-mortality associations were higher in the moderate season than in the hot and cold seasons for all countries/regions except for Thailand and Moldova. The effect estimates in the moderate season for mortality associated with an IQR increase in TV ranged from an increase of 0.39% in the United Kingdom to an increase of 1.45% in China. Table S2 shows the associations between TV and mortality on different exposure days in three seasons. Briefly, the effect estimates varied by exposure days; for example, when we used 0–1 days’ TV exposure, the cold season had higher effect estimates for TV-mortality associations than those in the hot and cold seasons in Brazil, China, and Thailand.

**Table 2:** Percent change (95% confidence interval) in mortality associated with an interquantile range for each community (IQR) increase in 0–7 days’ temperature variability (°C) in the cold season (4 coldest months), the hot season (4 hottest months), and the moderate season (the 4 months not included in the cold and hot seasons), after controlling for the main effect of temperature.

Country	Percent increase in mortality (%)		
	Cold season	Hot season	Moderate season
Australia	0.84 (–0.12, 1.82)	0.79 (0.20, 1.39)	0.85 (0.19, 1.51)
Brazil	0.47 (0.06, 0.89)	0.39 (–0.07, 0.84)	0.54 (0.14, 0.95)
Canada	0.57 (0.29, 0.85)	0.30 (0.06, 0.54)	0.61 (0.34, 0.88)
China	0.86 (0.18, 1.54)	0.93 (0.01, 1.86)	1.45 (0.49, 2.41)
Japan	0.72 (0.64, 0.80)	0.78 (0.70, 0.86)	1.08 (0.99, 1.16)
Korea	0.80 (0.47, 1.12)	0.85 (0.52, 1.19)	0.89 (0.57, 1.21)
Moldova	3.08 (–6.89, 14.11)	2.76 (–2.83, 8.67)	3.10 (–5.84, 12.88)
Spain	0.45 (0.16, 0.75)	0.49 (0.26, 0.72)	0.86 (0.60, 1.11)
Taiwan	0.20 (–0.36, 0.77)	0.20 (–0.14, 0.54)	0.86 (0.13, 1.61)
Thailand	0.14 (–0.50, 0.78)	0.27 (–0.39, 0.93)	0.26 (–0.09, 0.62)
United Kingdom	0.28 (0.10, 0.46)	0.34 (0.06, 0.62)	0.39 (0.14, 0.65)
United States	0.67 (0.55, 0.80)	0.47 (0.37, 0.56)	0.82 (0.71, 0.93)

**Figure 3:**
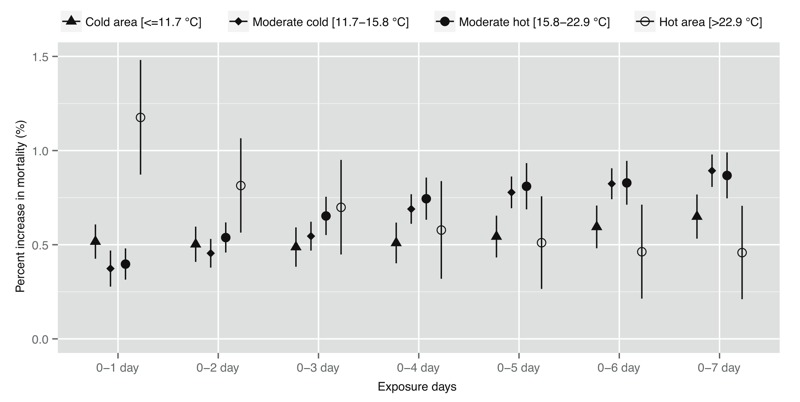
Percent change (95% confidence interval) in mortality associated with an interquantile range (for each community) increase in temperature variability (°C) on different exposure days in cold, moderate cold, moderate hot and hot areas, after controlling for the effect of daily mean temperature.

Figure 3 shows the pooled relationships between TV and mortality in cold, moderate cold, moderate hot, and hot areas. The effect estimates for TV-mortality associations were higher in hot areas than in cold, moderate cold, and moderate hot areas when using short exposure durations (0–1 and 0–2 days). However, the effect estimates were greater in moderate cold and moderate hot areas than in cold and hot areas when using longer exposure durations (0–5, 0–6, and 0–7 days).

Our results were robust to a changed lag structure of 28 days for temperature, modified degrees of freedom for temperature (3–6 df), and inclusion of relative humidity into the analyses (results not shown). When we included heat waves and cold spells into the models, the results were similar. When we calculated the effects per 1°C increase in TV (see Figure S2), the patterns (e.g., lag effect) of mortality risks did not change for any of the countries/regions, whereas the magnitude of mortality risks was lower than those using an IQR increase. Although replacing the daily mean temperature with the daily maximum and daily minimum temperatures in our model did not qualitatively change the evidence for an association of TV with mortality (see Figure S3), we could not control for daily maximum and daily minimum temperatures at the same time because they have very strong collinearity. Thus, we could not fully control for the residual confounding from extreme minimum and maximum temperatures, so it is also possible that the association of TV with mortality reflects adverse effects of the more extreme maximum or minimum temperatures that a high diurnal temperature range, and hence TV, would imply.

Our generalization of Granger causality analysis showed that there was no association between daily mortality and future 7 days’ TV exposure (see Figure S4). This finding confirms that there is little residual confounding in our models, and it strengthens the evidence for a cause–effect relationship between TV and mortality.

## Discussion

This study used consistent methods to examine TV-mortality associations for 372 communities across 12 countries/regions, including countries from both developing and developed regions with different climate patterns (i.e., tropical, subtropical, and temperate). We developed a novel method to calculate TV by taking into account both intraday and interday variability. We found that in all countries/regions, TV was associated with an increased risk of death, even after controlling for the main effects of temperature. The associations between TV and mortality appeared in different exposure durations in different countries. In general, TV-mortality associations varied with season. People were more sensitive to acute exposure to TV in warm areas than in cold areas, whereas they were more sensitive to long exposures to TV in cold areas than in hot areas.

Our findings are generally consistent with those of previous studies of intraday or interday TV (Lin et al. 2013; Qiu et al. 2013; Yang et al. 2013). However, those prior studies examined intraday and interday TV separately, and they did not consider the delayed effects of intraday or interday TV (Lin et al. 2013; Qiu et al. 2013; Yang et al. 2013). Additionally, they did not fully account for lagged effects of temperature, even though some studies controlled for the confounding effect of daily mean temperature on the day of death. In fact, the effects of cold temperatures include the lagged effects after several weeks of exposure, whereas the effects of hot temperatures relate to more recent days of exposure (Gasparrini and Armstrong 2013; Guo et al. 2011a). If the main effects of temperature are not fully controlled, the estimates of TV on mortality could be overestimated, as our findings revealed here (Figure 1).

Substantial evidence from the physiology literature has shown that people can have difficulty with thermoregulation and acclimatization to extreme cold and hot temperatures (Buguet 2007; Epstein and Moran 2006; Nixdorf-Miller et al. 2006) and that the automatic thermoregulation system cannot fully adapt to unstable weather (Kan et al. 2007; Liang et al. 2009). The thermoregulatory system of the human body might not respond efficiently to sudden changes (drops or increases) in temperature within a very short period of time (Martinez-Nicolas et al. 2015). People may feel uncomfortable with sudden intraday and interday changes in temperature because they are not well prepared for TV not only physiologically but also with regard to behavioral patterns (Garrett et al. 2009, 2011). Unstable temperatures have been shown to affect heart rate, blood pressure, blood cholesterol levels, plasma fibrinogen concentrations, peripheral vasoconstriction, platelet viscosity, autonomic control of the heart, and the immune system’s capability to resist infectious agents (Ballester et al. 1997; Carder et al. 2005; Garrett et al. 2009, 2011; Halonen et al. 2010, 2011a, 2011b; Martinez-Nicolas et al. 2015). These alterations may trigger cardiovascular and respiratory events. In addition, considering the characteristics of the present study, it is not possible to identify the groups most sensitive to TV. Most likely, older segments of the population may be most vulnerable to TV because of the progressive decrease in thermoregulatory ability associated with aging as well as the higher prevalence of comorbidities.

We found that different countries had different patterns of TV-mortality associations. For example, after controlling for the effects of daily mean temperature, the associations between TV and mortality were more acute in Brazil, Thailand, and the United Kingdom than in Australia, China, Japan, Korea, Spain, and the United States. Moreover, the evidence on the impacts of TV in different seasons is not consistent by country. Some countries had their highest effect estimates in the moderate season, whereas others had their highest effect estimates in the cold season or in the hot season. People living in hot areas were more sensitive to acute TV exposure than those in cold areas, whereas people living in moderate areas were more sensitive to long TV exposure than those in hot and cold areas. These differences may be caused by people adapting to their local climates via a range of physiological, behavioral, and technological adaptations (Nielsen et al. 1993). Clearly, further studies are needed to fully assess the reasons underlying the variations.

This study has several strengths. This is the first study to calculate TV that accounts for both intraday and interday variability. We developed a novel method to calculate TV using the standard deviation of the minimum and maximum temperatures of the current day and those of the preceding days. We used a large multi-country, multi-city data set and used consistent procedures and definitions. The same statistical method was used for each community, which makes it possible to compare the results directly. In addition, the TV-mortality associations were assessed in models after controlling for the nonlinear and delayed effects of daily temperature using flexible distributed lag nonlinear models. Additionally, a range of sensitivity analyses were performed to test the robustness of our results.

This study also has some limitations. As in other similar time-series studies, we used temperature data from fixed sites rather than individual exposures, which can create measurement errors in exposure to some extent. However, these measurement errors are likely to be random, which typically results in underestimation of the relative risks (Guo et al. 2013). There are insufficient data to address reasons for the variation in TV-mortality associations across communities. In addition, our data set did not include age- or cause-specific mortality, so we investigated only all-cause/nonaccidental mortality.

## Conclusions

Our findings provide strong evidence that TV is associated with an increased risk of mortality in different countries. These findings may have implications for assessing TV-related health risks and for developing public health policies to minimize the health consequences of unstable weather conditions. Our findings also suggest that projecting the health risks of climate change should take into account both the impacts of temperature increases and those of TV. However, more comprehensive epidemiological studies are needed across the globe because our results indicate that the health effects of TV vary greatly among different countries and climate zones.

## Supplemental Material

(896 KB) PDF

## References

[r1] Ballester F, Corella D, Pérez-Hoyos S, Sáez M, Hervás A (1997). Mortality as a function of temperature. A study in Valencia, Spain, 1991–1993.. Int J Epidemiol.

[r2] Basu R 2009 High ambient temperature and mortality: a review of epidemiological studies from 2001 to 2008. Environ Health 8 40, doi:10.1186/1476-069X-8-40 19758453 PMC2759912

[r3] Basu R, Samet JM (2002). Relation between elevated ambient temperature and mortality: a review of the epidemiologic evidence.. Epidemiol Rev.

[r4] Bhaskaran K, Gasparrini A, Hajat S, Smeeth L, Armstrong B (2013). Time series regression studies in environmental epidemiology.. Int J Epidemiol.

[r5] Buguet A (2007). Sleep under extreme environments: effects of heat and cold exposure, altitude, hyperbaric pressure and microgravity in space.. J Neurol Sci.

[r6] Carder M, McNamee R, Beverland I, Elton R, Cohen G, Boyd J (2005). The lagged effect of cold temperature and wind chill on cardiorespiratory mortality in Scotland.. Occup Environ Med.

[r7] Epstein Y, Moran DS (2006). Thermal comfort and the heat stress indices.. Ind Health.

[r8] Field CB (2012). Managing the Risks of Extreme Events and Disasters to Advance Climate Change Adaptation: Special Report of the Intergovernmental Panel on Climate Change..

[r9] Flanders WD, Klein M, Darrow LA, Strickland MJ, Sarnat SE, Sarnat JA (2011). A method for detection of residual confounding in time-series and other observational studies.. Epidemiology.

[r10] Garrett AT, Goosens NG, Rehrer NJ, Patterson MJ, Cotter JD (2009). Induction and decay of short-term heat acclimation.. Eur J Appl Physiol.

[r11] Garrett AT, Rehrer NJ, Patterson MJ (2011). Induction and decay of short-term heat acclimation in moderately and highly trained athletes.. Sports Med.

[r12] Gasparrini A Armstrong B 2013 Reducing and meta-analysing estimates from distributed lag non-linear models. BMC Med Res Methodol 13 1, doi:10.1186/1471-2288-13-1 23297754 PMC3599933

[r13] Gasparrini A, Armstrong B, Kenward MG (2010). Distributed lag non-linear models.. Stat Med.

[r14] Gasparrini A, Armstrong B, Kenward MG (2012). Multivariate meta-analysis for non-linear and other multi-parameter associations.. Stat Med.

[r15] Gasparrini A Guo Y Hashizume M Kinney PL Petkova EP Lavigne E 2015a Temporal variation in heat-mortality associations: a multi-country study. Environ Health Perspect 123 1200 1207, doi:10.1289/ehp.1409070 25933359 PMC4629745

[r16] Gasparrini A Guo Y Hashizume M Lavigne E Zanobetti A Schwartz J 2015b Mortality risk attributable to high and low ambient temperature: a multicountry observational study. Lancet 386 369 375, doi:10.1016/S0140-6736(14)62114-0 26003380 PMC4521077

[r17] Guo Y Barnett AG Pan X Yu W Tong S 2011a The impact of temperature on mortality in Tianjin, China: a case-crossover design with a distributed lag nonlinear model. Environ Health Perspect 119 1719 1725, doi:10.1289/ehp.1103598 21827978 PMC3261984

[r18] Guo Y, Barnett AG, Tong S (2013). Spatiotemporal model or time series model for assessing city-wide temperature effects on mortality?. Environ Res.

[r19] Guo Y Barnett AG Yu W Pan X Ye X Huang C 2011b A large change in temperature between neighbouring days increases the risk of mortality. Plos One 6 e16511, doi:10.1371/journal.pone.0016511 21311772 PMC3032790

[r20] Guo Y, Gasparrini A, Armstrong B, Li S, Tawatsupa B, Tobias A (2014). Global variation in the effects of ambient temperature on mortality: a systematic evaluation.. Epidemiology.

[r21] Halonen JI Zanobetti A Sparrow D Vokonas PS Schwartz J 2010 Associations between outdoor temperature and markers of inflammation: a cohort study. Environ Health 9 42, doi:10.1186/1476-069X-9-42 20653951 PMC2920265

[r22] Halonen JI, Zanobetti A, Sparrow D, Vokonas PS, Schwartz J (2011a). Outdoor temperature is associated with serum HDL and LDL.. Environ Res.

[r23] Halonen JI, Zanobetti A, Sparrow D, Vokonas PS, Schwartz J (2011b). Relationship between outdoor temperature and blood pressure.. Occup Environ Med.

[r24] Kan H, London SJ, Chen H, Song G, Chen G, Jiang L (2007). Diurnal temperature range and daily mortality in Shanghai, China.. Environ Res.

[r25] Liang WM, Liu WP, Kuo HW (2009). Diurnal temperature range and emergency room admissions for chronic obstructive pulmonary disease in Taiwan.. Int J Biometeorol.

[r26] Lin H Zhang Y Xu Y Xu X Liu T Luo Y 2013 Temperature changes between neighboring days and mortality in summer: a distributed lag non-linear time series analysis. PLoS One 8 e66403, doi:10.1371/journal.pone.0066403 23826095 PMC3691212

[r27] Martinez-Nicolas A, Meyer M, Hunkler S, Madrid JA, Rol MA, Meyer AH (2015). Daytime variation in ambient temperature affects skin temperatures and blood pressure: ambulatory winter/summer comparison in healthy young women.. Physiol Behav.

[r28] Nielsen B, Hales JR, Strange S, Christensen NJ, Warberg J, Saltin B (1993). Human circulatory and thermoregulatory adaptations with heat acclimation and exercise in a hot, dry environment.. J Physiol.

[r29] Nixdorf-Miller A, Hunsaker DM, Hunsaker JC (2006). Hypothermia and hyperthermia medicolegal investigation of morbidity and mortality from exposure to environmental temperature extremes.. Arch Pathol Lab Med.

[r30] Qiu H, Yu IT, Tse LA, Tian L, Wang X, Wong TW (2013). Is greater temperature change within a day associated with increased emergency hospital admissions for heart failure?. Circ Heart Fail.

[r31] Samet JM, Dominici F, Curriero FC, Coursac I, Zeger SL (2000). Fine particulate air pollution and mortality in 20 U.S. cities, 1987–1994.. N Engl J Med.

[r32] Shi L, Kloog I, Zanobetti A, Liu PF, Schwartz JD (2015). Impacts of temperature and its variability on mortality in New England.. Nat Clim Chang.

[r33] Stocker TF (2014). Climate Change 2013: The Physical Science Basis: Working Group I Contribution to the Fifth Assessment Report of the Intergovernmental Panel on Climate Change..

[r34] Yang J, Liu HZ, Ou CQ, Lin GZ, Zhou Q, Shen GC (2013). Global climate change: impact of diurnal temperature range on mortality in Guangzhou, China.. Environ Pollut.

[r35] Ye X Wolff R Yu W Vaneckova P Pan X Tong S 2012 Ambient temperature and morbidity: a review of epidemiological evidence. Environ Health Perspect 120 19 28, doi:10.1289/ehp.1003198 21824855 PMC3261930

[r36] Zanobetti A, O’Neill MS, Gronlund CJ, Schwartz JD (2012). Summer temperature variability and long-term survival among elderly people with chronic disease.. Proc Natl Acad Sci U S A.

