# Supplementary material for: Temperature Variability and Mortality: A Multi-Country Study
Source: Environ Health Perspect. 2016 Jun 3;124(10):1554–9. doi: 10.1289/EHP149 (PMC5047764; doi:10.1289/EHP149)
Supplement: (896 KB) PDF [file EHP149.s001.acco.pdf]

Environ Health Perspect
DOI: 10.1289/EHP149

Note to readers with disabilities: *EHP* strives to ensure that all journal content is accessible to all readers. However, some figures and Supplemental Material published in *EHP* articles may not conform to 508 standards due to the complexity of the information being presented. If you need assistance accessing journal content, please contact ehp508@niehs.nih.gov. Our staff will work with you to assess and meet your accessibility needs within 3 working days.

Supplemental Material

Temperature Variability and Mortality: A Multi-Country Study

Yuming Guo, Antonio Gasparrini, Ben G. Armstrong, Benjawan Tawatsupa, Aurelio Tobias, Eric Lavigne, Micheline de Sousa Zanotti Stagliorio Coelho, Xiaochuan Pan, Ho Kim, Masahiro Hashizume, Yasushi Honda, Yue Leon Guo, Chang-Fu Wu, Antonella Zanobetti, Joel D. Schwartz, Michelle L. Bell, Ala Overcenco, Kornwipa Punnasiri, Shanshan Li, Linwei Tian, Paulo Saldiva, Gail Williams, and Shilu Tong

Table of Contents

Data Collection

- Australia
- Brazil
- Canada
- China
- Japan
- Korea
- Moldova
- Spain
- Taiwan
- Thailand data
- UK data
- USA data

Table S1. Summary statistics for period, deaths, mean temperature, and temperature variability (from 0–1 day to 0–7 day's exposure) in 372 communities in 12 countries/regions.

Table S2. Percent change (mean and 95% CI) of mortality associated with an IQR (inter-quartile range) increase in temperature variability (°C) on different exposure days in cold (four coldest months), hot (four hottest months) and moderate (except four coldest and four hottest seasons) seasons, after controlling for main effect of temperature.

Figure S1. Locations of communities stratified by cold, moderate cold, moderate hot and hot areas, by the quantiles ($\leq 25^{th}$, 25th–50th, 50th –75th, and $>75^{th}$) of their annual mean temperatures (each community has one value of annual mean temperature).

Figure S2. Percent change (95% CI) in mortality associated with one degree increase in temperature variability (°C) on different exposure days, (A) after controlling for the effect of daily mean temperature, (B) without controlling for the effect of temperature.

Figure S3. Percent change (95% CI) in mortality associated with an IQR (for each community) increase in temperature variability (°C) on different exposure days, (A) after controlling for the effect of daily minimum temperature, (B) controlling for the effect of daily maximum temperature.

Figure S4. Percent change (95% CI) in mortality associated with future 7 days' temperature variability (°C), after controlling for the main effect of temperature, seasonality, and day of the week.

Data Collection

Australia

We collected data from Melbourne, Sydney and Brisbane between 1st of January 1988 and 31st of May 2009. Daily mortality, obtained from the Australian Bureau of Statistics, is represented by counts of deaths for non-external causes only (ICD-9: 0-799; ICD-10: A00-R99). Daily minimum, mean (24-hour average) and maximum temperature (in ˚C) and relative humidity (in %) were obtained from the Australian Bureau of Meteorology. We selected all available meteorological stations located within ≤30 km of each city's Central Business District (CBD) (7 stations in Brisbane, 7 stations in Melbourne and 11 stations in Sydney). We calculated the daily averages of climatic variables using all records from meteorological stations in each city. When there was a missing value (≤1.3%) for a particular meteorological station, observations recorded from the remaining weather stations were used to compute the daily average values.

Brazil

Data on daily deaths for non-external causes only (ICD-10 codes: A00-R99) in 18 cities (see full list in Table S1 below) between 1st of January 1997 and 31st of December 2011 were collected from Ministry of Health, Brazil. Data on mean daily temperature (computed as the 24-hours average based on hourly measurements) and relative humidity were obtained during the same study period from Weather Meteorological Service of Brazil.

Canada

We obtained daily data on non-accidental mortality from Statistics Canada through access to the Canadian Mortality Database for the period of 1986 to 2009 for 21 census metropolitan areas (CMA). Daily meteorological data were obtained from Environment Canada using the airport monitoring station located closest to the CMA centre. Daily averages of temperature and relative humidity were computed based on hourly measurements.

China

We obtained daily data from 6 cities (Hong Kong, Beijing, Tianjin, Shanghai, Wuhan, and Guangzhou) in China. Daily data on non-accidental mortality in urban areas of Beijing, Tianjin, Shanghai, and Guangzhou were obtained from the China Information System for Death Register and Report of Chinese Centre for Disease Control and Prevention (China CDC) from 2004 to 2008. Daily weather data were obtained from the China Meteorological Data Sharing Service System for each city.

For Hong Kong data, daily non-accidental mortality data were obtained from the Hong Kong Census and Statistics Department during 2002–2009. Daily minimum, mean, and maximum temperatures and relative humidity data were obtained from the Hong Kong Observatory for the same period.

Japan

Data on daily deaths for non-external causes only (ICD-9 codes: 1-799; ICD-10 codes: A00-R99) in 47 prefectures (see full list in Table S1 below) between 1st of January 1972 and 31st of December 2012 were collected. Data on daily minimum, mean (computed as the 24-hours

average based on hourly measurements) and maximum temperatures and relative humidity were obtained for the same study period.

Korea

Data on daily deaths for non-external causes only (ICD-9 codes: 1-799; ICD-10 codes: A00-R99) in 7 cities (see full list in Table S1 below) between 1st of January 1992 and 31st of December 2010 were collected. Data on daily minimum, mean (computed as the 24-hours average based on hourly measurements) and maximum temperatures and relative humidity were obtained for the same study period.

Moldova

Data on daily deaths for non-external causes only (ICD-10 codes: A00-R99) in 4 cities (see full list in Table S1 below) between 1st of January 2001 and 31st of December 2010 were collected from National Centre of Public Health Management. Data on daily minimum, mean (computed as the 24-hours average based on hourly measurements) and maximum temperatures and relative humidity were obtained by State Hydrometeorological Service for the same study period.

Spain

We obtained daily data on non-accidental causes for the 51 capital cities from the Spain National Institute of Statistics for summer months (from 1st June to 30th September) from 1990 to 2010. Daily minimum, mean and maximum temperatures for the 51 capital cities were collected from the Spain National Meteorology Agency for the same study period. We did not get the data on relative humidity, because it is not available.

Taiwan

Data on daily deaths for non-external causes (ICD-9 codes: 1-799; ICD-10 codes: A00-R99) in Kaohsiung, Taipei and Taichung between 1st of January 1994 and 31st of December 2007 were collected. Data on daily minimum, mean (computed as the 24-hours average based on hourly measurements) and maximum temperatures and relative humidity were obtained for the same study period.

Thailand data

We obtained daily data on non-accidental deaths from the Ministry of Public Health, Thailand for 62 provinces during 1999–2008. The daily weather data (daily minimum, mean, and maximum temperatures and mean relative humidity) were obtained from the Meteorological Department, Ministry of Information and Communication Technology. There were 117 weather stations in 62 provinces, with at least one weather monitoring station in each province.

UK data

We obtained daily data on non-accidental mortality from the Office of National Statistics during 1993–2006. Records include the date of death and postcode of residence at time of death. The postcodes were used to divide deaths into 10 government regions and date to make daily series of counts for each region. The daily weather data (daily minimum, mean, and maximum temperatures and mean relative humidity) were downloaded from the British Atmospheric Data Centre. There was a mean of 29 stations contributing data to each regional series, from a minimum of 7 in London to a maximum of 44 in Wales.

USA data

We collected data from 135 cities (see full list in Table S1) between 1st of January 1985 and 31st of December 2006. Daily mortality, obtained from the National Center for Health Statistics (NCHS), is represented by counts of deaths for non-external causes only (ICD-9: 0-799; ICD-10: A00-R99). Daily minimum, mean (in ˚C, computed as the 24-hour average based on hourly measurements) and maximum temperatures and relative humidity (in %, computed from the 24-h average of hourly measurements of dew point temperature) were obtained from the National Climatic Data Center (NCDC) of the National Oceanic and Atmospheric Administration (NOAA). A single weather station was selected for each city in the land based station data or NCDC, based on the proximity to the city's population centre. In 6 cities where multiple observations were missing from all the nearby monitors, hourly data from the Integrated Surface Database Lite of NCDC were converted in daily values. For 25 stations missing dew point data, dew point data were obtained from the nearest station with dew point data.

Table S1: Summary statistics for period, deaths, mean temperature, and temperature variability (from 0–1 day to 0–7 day's exposure) in 372 communities in 12 countries/regions.

Community	Period	Mean death	Average temperature	Mean (IQR) of temperature variability						
				0–1day	0–2day	0–3day	0–4day	0–5day	0–6day	0–7day
MELBOURNE, AUSTRALIA	1988-2009	58	15.7	5.2 (2.5)	5.1 (2.4)	5.1 (2.3)	5.2 (2.2)	5.2 (2.1)	5.2 (2.0)	5.2 (2.0)
SYDNEY, AUSTRALIA	1988-2009	69	18.3	4.5 (2.0)	4.4 (1.7)	4.4 (1.6)	4.4 (1.5)	4.4 (1.4)	4.4 (1.3)	4.4 (1.3)
BRISBANE, AUSTRALIA	1988-2009	25	20.3	5.3 (2.1)	5.1 (2.0)	5.0 (1.9)	5.0 (1.8)	5.0 (1.8)	5.0 (1.7)	4.9 (1.7)
PORTO ALEGRE, BRAZIL	1997-2011	39	19.7	5.8 (2.5)	5.6 (2.0)	5.6 (1.7)	5.6 (1.5)	5.6 (1.4)	5.7 (1.3)	5.7 (1.3)
CURITIBA, BRAZIL	1997-2011	28	17.7	6.1 (2.3)	5.9 (1.9)	5.9 (1.7)	5.9 (1.6)	5.9 (1.5)	5.9 (1.4)	5.9 (1.3)
SAP PAULO, BRAZIL	1997-2011	178	20.3	5.9 (2.1)	5.7 (1.7)	5.6 (1.5)	5.6 (1.4)	5.6 (1.3)	5.6 (1.2)	5.6 (1.2)
VITORIA, BRAZIL	1997-2011	9	24.8	4.3 (1.2)	4.1 (1.0)	4.1 (0.8)	4.1 (0.8)	4.1 (0.7)	4.1 (0.7)	4.1 (0.6)
BELO HORIZONTE, BRAZIL	1997-2011	88	22	5.4 (1.4)	5.2 (1.2)	5.1 (1.1)	5.1 (1.0)	5.1 (0.9)	5.0 (0.9)	5.0 (0.9)
GOIANIA, BRAZIL	1997-2011	25	24.5	7.3 (2.4)	7.0 (2.2)	6.8 (2.2)	6.7 (2.1)	6.7 (2.1)	6.7 (2.1)	6.6 (2.0)
BRASILIA, BRAZIL	1997-2011	21	21.3	5.8 (1.6)	5.5 (1.5)	5.4 (1.4)	5.3 (1.4)	5.3 (1.4)	5.3 (1.3)	5.3 (1.3)
CUIABA, BRAZIL	1997-2011	9	26.4	6.9 (2.7)	6.6 (2.5)	6.5 (2.4)	6.5 (2.4)	6.5 (2.4)	6.4 (2.3)	6.4 (2.3)
SALVADOR, BRAZIL	1997-2011	41	25.6	3.8 (1.1)	3.6 (1.0)	3.5 (1.0)	3.5 (0.9)	3.5 (0.9)	3.5 (0.9)	3.4 (0.9)
MACEIO, BRAZIL	1997-2011	17	25.2	5.2 (1.3)	4.9 (1.2)	4.8 (1.1)	4.8 (1.1)	4.7 (1.0)	4.7 (1.0)	4.7 (1.0)
RECIFE, BRAZIL	1997-2011	48	26	4.2 (1.1)	4.0 (0.9)	3.9 (0.8)	3.9 (0.7)	3.9 (0.7)	3.8 (0.7)	3.8 (0.6)
JOAO PESSOA, BRAZIL	1997-2011	14	27	3.6 (1.0)	3.4 (0.8)	3.4 (0.8)	3.3 (0.7)	3.3 (0.7)	3.3 (0.7)	3.3 (0.6)
NATAL, BRAZIL	1997-2011	15	19.7	5.8 (2.5)	5.6 (2.0)	5.6 (1.7)	5.6 (1.5)	5.6 (1.4)	5.7 (1.3)	5.7 (1.3)
TERESINA, BRAZIL	1997-2011	12	27.4	6.2 (2.4)	5.9 (2.2)	5.8 (2.2)	5.7 (2.1)	5.7 (2.1)	5.7 (2.1)	5.6 (2.1)
FORTALEZA, BRAZIL	1997-2011	37	27	4.2 (0.7)	4.0 (0.6)	3.9 (0.5)	3.8 (0.5)	3.8 (0.5)	3.8 (0.5)	3.8 (0.5)
MANAUS, BRAZIL	1997-2011	18	27.2	5.2 (1.3)	5.0 (1.1)	4.9 (1.1)	4.8 (1.0)	4.8 (1.0)	4.8 (1.0)	4.8 (1.0)
SAO LUIS, BRAZIL	1997-2011	15	26.9	4.3 (0.8)	4.1 (0.7)	4.0 (0.7)	4.0 (0.6)	4.0 (0.6)	3.9 (0.6)	3.9 (0.6)
BELEM, BRAZIL	1997-2011	24	26.9	5.4 (1.0)	5.1 (0.9)	5.0 (0.9)	4.9 (0.9)	4.9 (0.8)	4.8 (0.8)	4.8 (0.8)
NARATHIWAT, THAILAND	1999-2008	4	27.7	5.9 (2.0)	5.7 (1.9)	5.5 (1.9)	5.5 (1.8)	5.4 (1.8)	5.4 (1.8)	5.4 (1.8)
YALA, THAILAND	1999-2008	3	27.6	4.8 (1.2)	4.6 (1.1)	4.5 (1.0)	4.4 (1.0)	4.4 (0.9)	4.3 (0.9)	4.3 (0.9)

PATTANI, THAILAND	1999-2008	3	27.8	5.3 (1.8)	5.1 (1.5)	5.0 (1.4)	4.9 (1.3)	4.9 (1.3)	4.9 (1.2)	4.9 (1.1)
SONGKHLA, THAILAND	1999-2008	9	27.9	5.0 (1.2)	4.8 (1.1)	4.7 (1.0)	4.6 (1.0)	4.6 (1.0)	4.6 (0.9)	4.6 (0.9)
TRANG, THAILAND	1999-2008	4	28.7	4.7 (1.6)	4.5 (1.4)	4.4 (1.4)	4.3 (1.3)	4.3 (1.3)	4.3 (1.3)	4.3 (1.3)
KRABI, THAILAND	1999-2008	2	27.8	4.8 (1.6)	4.5 (1.4)	4.5 (1.4)	4.4 (1.3)	4.4 (1.3)	4.4 (1.3)	4.3 (1.3)
NAKHON SI THAMMARAT, THAILAND	1999-2008	11	27.3	5.1 (1.3)	4.9 (1.2)	4.8 (1.1)	4.7 (1.0)	4.7 (1.0)	4.7 (1.0)	4.7 (1.0)
SURAT THANI, THAILAND	1999-2008	6	27.3	5.8 (2.6)	5.6 (2.4)	5.5 (2.4)	5.4 (2.4)	5.4 (2.4)	5.4 (2.4)	5.4 (2.4)
CHUMPHON, THAILAND	1999-2008	3	28.6	6.0 (1.9)	5.7 (1.7)	5.6 (1.7)	5.5 (1.7)	5.5 (1.7)	5.4 (1.6)	5.4 (1.6)
PRACHUAP KHIRI KHAN, THAILAND	1999-2008	4	27.9	5.6 (1.4)	5.4 (1.2)	5.3 (1.1)	5.2 (1.0)	5.2 (1.0)	5.1 (0.9)	5.1 (0.9)
CHANTHABURI, THAILAND	1999-2008	6	27.3	5.1 (1.5)	4.9 (1.3)	4.8 (1.2)	4.7 (1.2)	4.7 (1.1)	4.7 (1.0)	4.7 (1.0)
RAYONG, THAILAND	1999-2008	5	25.8	6.4 (3.7)	6.1 (3.5)	6.0 (3.4)	5.9 (3.4)	5.8 (3.3)	5.8 (3.3)	5.8 (3.3)
PHETCHABURI, THAILAND	1999-2008	4	28	5.2 (1.1)	5.0 (1.0)	4.8 (0.9)	4.8 (0.8)	4.8 (0.8)	4.7 (0.8)	4.7 (0.8)
CHON BURI, THAILAND	1999-2008	13	28.4	3.8 (1.3)	3.6 (1.2)	3.6 (1.1)	3.5 (1.0)	3.5 (1.0)	3.5 (1.0)	3.5 (1.0)
SAMUTPRAKAN, THAILAND	1999-2008	8	28.3	2.5 (1.3)	2.5 (1.1)	2.4 (1.0)	2.4 (0.9)	2.4 (0.9)	2.4 (0.9)	2.4 (0.8)
RATCHABURI, THAILAND	1999-2008	8	26.8	7.1 (3.4)	6.8 (3.2)	6.6 (3.1)	6.6 (3.1)	6.5 (3.1)	6.5 (3.1)	6.5 (3.1)
SAMUT SAKHON, THAILAND	1999-2008	4	28.2	4.3 (1.1)	4.1 (0.9)	4.0 (0.9)	4.0 (0.8)	4.0 (0.8)	4.0 (0.7)	3.9 (0.7)
CHACHOENGSAO, THAILAND	1999-2008	5	26.1	6.6 (3.2)	6.3 (3.0)	6.2 (2.9)	6.1 (2.9)	6.1 (2.9)	6.1 (2.9)	6.1 (2.9)
BANGKOK, THAILAND	1999-2008	66	29.3	4.7 (0.9)	4.5 (0.8)	4.4 (0.8)	4.4 (0.7)	4.3 (0.7)	4.3 (0.7)	4.3 (0.6)
SA KAEO, THAILAND	1999-2008	4	27.7	5.1 (1.4)	4.9 (1.2)	4.8 (1.2)	4.8 (1.1)	4.7 (1.1)	4.7 (1.0)	4.7 (1.0)
NONTHABURI, THAILAND	1999-2008	8	27.8	6.5 (2.7)	6.2 (2.5)	6.0 (2.5)	6.0 (2.4)	5.9 (2.5)	5.9 (2.4)	5.9 (2.4)
NAKHON PATHOM, THAILAND	1999-2008	7	27.9	6.0 (2.0)	5.7 (1.8)	5.6 (1.8)	5.5 (1.8)	5.5 (1.8)	5.5 (1.8)	5.5 (1.8)
KANCHANABURI, THAILAND	1999-2008	6	28.4	5.9 (1.6)	5.6 (1.4)	5.5 (1.4)	5.5 (1.3)	5.4 (1.3)	5.4 (1.3)	5.4 (1.3)
PATHUM THANI, THAILAND	1999-2008	6	27	6.1 (2.5)	5.8 (2.3)	5.7 (2.3)	5.7 (2.2)	5.6 (2.3)	5.6 (2.3)	5.6 (2.3)
PRACHIN BURI, THAILAND	1999-2008	4	28.4	4.2 (1.1)	4.0 (1.0)	3.9 (0.9)	3.9 (0.9)	3.8 (0.9)	3.8 (0.9)	3.8 (0.8)
AYUTTHAYA, THAILAND	1999-2008	7	28.4	6.0 (1.7)	5.7 (1.6)	5.6 (1.6)	5.5 (1.5)	5.5 (1.6)	5.5 (1.6)	5.4 (1.5)
SUPHANBURI, THAILAND	1999-2008	7	28.1	5.7 (1.9)	5.5 (1.8)	5.4 (1.7)	5.3 (1.6)	5.3 (1.6)	5.3 (1.6)	5.2 (1.6)
SARABURI, THAILAND	1999-2008	6	27.9	5.5 (1.9)	5.2 (1.8)	5.1 (1.8)	5.1 (1.7)	5.0 (1.7)	5.0 (1.7)	5.0 (1.7)
LOP BURI, THAILAND	1999-2008	8	27.3	4.6 (1.3)	4.4 (1.1)	4.3 (1.1)	4.2 (1.1)	4.2 (1.1)	4.2 (1.1)	4.2 (1.1)
SURIN, THAILAND	1999-2008	8	27.5	6.0 (2.2)	5.8 (2.1)	5.6 (2.1)	5.6 (2.1)	5.6 (2.1)	5.5 (2.1)	5.5 (2.1)
NAKHON RATCHASIMA, THAILAND	1999-2008	20	27.1	5.8 (2.0)	5.5 (1.9)	5.4 (1.9)	5.4 (1.8)	5.3 (1.8)	5.3 (1.8)	5.3 (1.8)

BURI RAM, THAILAND	1999-2008	9	26.9	6.1 (2.1)	5.8 (2.0)	5.7 (2.0)	5.6 (2.0)	5.6 (2.0)	5.6 (2.0)	5.6 (2.0)
SI SA KET, THAILAND	1999-2008	10	27.3	5.9 (2.5)	5.7 (2.4)	5.6 (2.3)	5.5 (2.3)	5.5 (2.3)	5.5 (2.3)	5.4 (2.3)
UBON RATCHATHANI, THAILAND	1999-2008	13	27.5	6.3 (2.5)	6.0 (2.4)	5.9 (2.3)	5.8 (2.3)	5.8 (2.3)	5.8 (2.3)	5.7 (2.3)
NAKHON SAWAN, THAILAND	1999-2008	10	28.5	5.9 (1.7)	5.7 (1.6)	5.5 (1.6)	5.5 (1.6)	5.5 (1.6)	5.4 (1.6)	5.4 (1.6)
YASOTHON, THAILAND	1999-2008	4	25.8	6.7 (3.4)	6.4 (3.1)	6.3 (3.0)	6.2 (3.0)	6.2 (3.0)	6.2 (3.0)	6.1 (3.0)
CHAIYAPHUM, THAILAND	1999-2008	8	27.8	5.9 (2.1)	5.6 (2.0)	5.5 (2.0)	5.5 (1.9)	5.5 (1.9)	5.4 (1.9)	5.4 (1.9)
AMNAT CHAROEN, THAILAND	1999-2008	3	27.5	4.6 (1.3)	4.4 (1.1)	4.3 (1.1)	4.2 (1.0)	4.2 (1.0)	4.2 (1.0)	4.2 (1.0)
ROI ET, THAILAND	1999-2008	11	27.1	5.8 (2.6)	5.5 (2.5)	5.5 (2.5)	5.4 (2.5)	5.4 (2.5)	5.4 (2.5)	5.4 (2.5)
MAHA SARAKHAM, THAILAND	1999-2008	7	28.8	5.6 (1.4)	5.4 (1.3)	5.3 (1.3)	5.2 (1.3)	5.2 (1.2)	5.1 (1.2)	5.1 (1.2)
PHETCHABUN, THAILAND	1999-2008	7	27.1	5.0 (1.3)	4.7 (1.2)	4.6 (1.0)	4.6 (1.0)	4.5 (0.9)	4.5 (0.9)	4.5 (0.8)
KALASIN, THAILAND	1999-2008	8	27.7	6.9 (2.2)	6.6 (2.1)	6.5 (2.0)	6.4 (2.0)	6.4 (2.0)	6.3 (2.0)	6.3 (2.0)
KHON KAEN, THAILAND	1999-2008	16	27.2	6.2 (2.5)	6.0 (2.4)	5.8 (2.3)	5.8 (2.3)	5.8 (2.3)	5.7 (2.3)	5.7 (2.3)
PHICHIT, THAILAND	1999-2008	4	27.8	5.6 (2.2)	5.3 (2.0)	5.2 (2.0)	5.1 (1.9)	5.1 (1.9)	5.1 (1.9)	5.1 (1.9)
MUKDAHAN, THAILAND	1999-2008	2	27.8	4.8 (2.1)	4.6 (1.9)	4.5 (1.8)	4.4 (1.7)	4.4 (1.7)	4.4 (1.7)	4.4 (1.7)
KAMPHAENG PHET, THAILAND	1999-2008	4	26.4	6.0 (2.8)	5.8 (2.6)	5.7 (2.6)	5.7 (2.6)	5.6 (2.6)	5.6 (2.6)	5.6 (2.6)
PHITSANULOK, THAILAND	1999-2008	9	28.3	6.0 (1.6)	5.7 (1.5)	5.6 (1.5)	5.5 (1.4)	5.5 (1.4)	5.5 (1.4)	5.5 (1.4)
TAK, THAILAND	1999-2008	3	28	4.7 (1.4)	4.5 (1.2)	4.4 (1.2)	4.4 (1.1)	4.3 (1.1)	4.3 (1.1)	4.3 (1.1)
SUKHOTHAI, THAILAND	1999-2008	5	28.3	5.7 (1.8)	5.5 (1.7)	5.4 (1.7)	5.3 (1.6)	5.3 (1.6)	5.3 (1.6)	5.2 (1.6)
SAKON NAKHON, THAILAND	1999-2008	9	26.5	6.0 (2.9)	5.7 (2.8)	5.6 (2.7)	5.6 (2.7)	5.6 (2.7)	5.5 (2.7)	5.5 (2.8)
NONG BUA LAM PHU, THAILAND	1999-2008	3	28.1	6.4 (2.4)	6.1 (2.2)	6.0 (2.2)	5.9 (2.1)	5.9 (2.2)	5.8 (2.2)	5.8 (2.1)
NAKHON PHANOM, THAILAND	1999-2008	4	27.8	5.2 (1.2)	4.9 (1.1)	4.8 (1.0)	4.8 (1.0)	4.7 (0.9)	4.7 (0.9)	4.7 (0.9)
UDON THANI, THAILAND	1999-2008	12	27.1	6.3 (2.7)	6.0 (2.5)	5.9 (2.5)	5.8 (2.5)	5.8 (2.5)	5.8 (2.5)	5.8 (2.5)
UTTARADIT, THAILAND	1999-2008	5	27.3	6.3 (2.7)	6.0 (2.6)	5.9 (2.5)	5.8 (2.5)	5.8 (2.5)	5.8 (2.5)	5.8 (2.5)
NONG KHAI, THAILAND	1999-2008	6	27.9	5.8 (1.6)	5.5 (1.4)	5.4 (1.3)	5.3 (1.3)	5.3 (1.3)	5.3 (1.2)	5.3 (1.3)
PHRAE, THAILAND	1999-2008	6	28.1	5.5 (1.6)	5.2 (1.4)	5.1 (1.4)	5.1 (1.3)	5.0 (1.3)	5.0 (1.3)	5.0 (1.3)
LAMPANG, THAILAND	1999-2008	10	28	5.9 (2.4)	5.6 (2.2)	5.5 (2.2)	5.5 (2.2)	5.4 (2.2)	5.4 (2.2)	5.4 (2.2)
LAMPHUN, THAILAND	1999-2008	4	26.2	6.9 (3.9)	6.6 (3.8)	6.5 (3.7)	6.4 (3.6)	6.4 (3.6)	6.4 (3.5)	6.4 (3.5)
NAN, THAILAND	1999-2008	5	26.6	7.0 (3.7)	6.7 (3.5)	6.6 (3.4)	6.5 (3.3)	6.5 (3.3)	6.5 (3.3)	6.4 (3.3)
CHIANG MAI, THAILAND	1999-2008	21	26.3	6.7 (3.4)	6.4 (3.2)	6.3 (3.1)	6.2 (3.0)	6.2 (3.0)	6.1 (3.0)	6.1 (3.0)

PHAYAO, THAILAND	1999-2008	7	27.2	6.4 (3.2)	6.1 (3.1)	6.0 (3.0)	5.9 (3.0)	5.9 (3.0)	5.9 (3.0)	5.8 (3.0)
CHIANG RAI, THAILAND	1999-2008	14	25.1	6.3 (3.8)	6.1 (3.5)	5.9 (3.4)	5.9 (3.4)	5.9 (3.4)	5.8 (3.3)	5.8 (3.3)
HONG KONG, CHINA	2002-2009	99	24.2	3.2 (1.0)	3.1 (0.8)	3.2 (0.8)	3.2 (0.7)	3.2 (0.7)	3.3 (0.7)	3.3 (0.7)
GUANGZHOU, CHINA	2004-2008	29	23.1	4.5 (1.6)	4.5 (1.3)	4.5 (1.2)	4.5 (1.1)	4.6 (1.1)	4.6 (1.1)	4.6 (1.1)
WUHAN, CHINA	2004-2008	12	18.2	4.6 (2.0)	4.5 (1.7)	4.5 (1.5)	4.6 (1.4)	4.6 (1.4)	4.6 (1.3)	4.7 (1.3)
SHANGHAI, CHINA	2004-2008	67	17.8	4.2 (2.2)	4.1 (1.9)	4.2 (1.8)	4.2 (1.7)	4.3 (1.7)	4.3 (1.7)	4.3 (1.6)
TIANJIN, CHINA	2004-2008	56	13.3	6.1 (2.5)	5.9 (2.1)	5.8 (1.9)	5.8 (1.8)	5.8 (1.7)	5.8 (1.7)	5.8 (1.7)
BEIJING, CHINA	2004-2008	74	13.6	6.0 (2.4)	5.8 (2.0)	5.7 (1.9)	5.7 (1.8)	5.7 (1.7)	5.7 (1.7)	5.7 (1.6)
KAOHSIUNG, TAIWAN	1994-2007	37	25.2	4.0 (1.1)	3.9 (1.0)	3.8 (1.0)	3.8 (1.0)	3.8 (1.0)	3.8 (1.0)	3.8 (1.0)
TAIPEI, TAIWAN	1994-2007	70	23.2	3.9 (2.0)	3.9 (1.6)	3.9 (1.4)	4.0 (1.3)	4.0 (1.2)	4.0 (1.2)	4.0 (1.1)
TAICHUNG, TAIWAN	1994-2007	28	23.6	4.9 (1.4)	4.7 (1.2)	4.7 (1.1)	4.6 (1.1)	4.6 (1.0)	4.6 (1.1)	4.6 (1.1)
GWANGJU, KOREA	1992-2010	14	14.1	5.3 (2.5)	5.2 (2.1)	5.2 (1.9)	5.2 (1.8)	5.2 (1.8)	5.2 (1.7)	5.2 (1.7)
BUSAN, KOREA	1992-2010	43	14.9	4.2 (1.6)	4.1 (1.5)	4.1 (1.4)	4.1 (1.4)	4.1 (1.4)	4.2 (1.4)	4.2 (1.4)
ULSAN, KOREA	1992-2010	9	14.5	5.2 (2.3)	5.1 (1.9)	5.1 (1.8)	5.1 (1.7)	5.2 (1.7)	5.2 (1.6)	5.2 (1.5)
DAEGU, KOREA	1992-2010	26	14.4	5.6 (2.3)	5.4 (1.9)	5.4 (1.8)	5.4 (1.6)	5.4 (1.6)	5.4 (1.5)	5.4 (1.5)
DAEJEON, KOREA	1992-2010	13	13	5.7 (2.5)	5.6 (2.2)	5.5 (2.0)	5.5 (1.9)	5.6 (1.8)	5.6 (1.7)	5.6 (1.7)
INCHEON, KOREA	1992-2010	24	12.5	4.4 (1.6)	4.3 (1.4)	4.3 (1.3)	4.3 (1.3)	4.4 (1.2)	4.4 (1.2)	4.4 (1.2)
SEOUL, KOREA	1992-2010	91	12.8	4.7 (1.7)	4.7 (1.4)	4.7 (1.3)	4.7 (1.3)	4.7 (1.3)	4.8 (1.2)	4.8 (1.2)
AICHI, JAPAN	1972-2012	104	15.7	5.3 (1.7)	5.1 (1.3)	5.1 (1.2)	5.1 (1.1)	5.1 (1.1)	5.1 (1.0)	5.1 (1.0)
AKITA, JAPAN	1972-2012	28	11.6	4.6 (2.2)	4.5 (1.9)	4.5 (1.6)	4.5 (1.5)	4.5 (1.5)	4.5 (1.4)	4.5 (1.4)
AOMORI, JAPAN	1972-2012	31	10.3	4.9 (2.4)	4.8 (2.1)	4.8 (1.8)	4.8 (1.7)	4.8 (1.6)	4.8 (1.5)	4.8 (1.5)
CHIBA, JAPAN	1972-2012	83	15.7	4.5 (1.5)	4.4 (1.3)	4.4 (1.2)	4.4 (1.1)	4.4 (1.0)	4.4 (1.0)	4.4 (1.0)
EHIME, JAPAN	1972-2012	33	16.3	5.0 (1.6)	4.8 (1.3)	4.8 (1.2)	4.8 (1.1)	4.8 (1.1)	4.8 (1.0)	4.8 (1.0)
FUKUSHIMA, JAPAN	1972-2012	44	13	5.5 (2.3)	5.4 (1.9)	5.4 (1.7)	5.4 (1.6)	5.4 (1.5)	5.4 (1.4)	5.4 (1.4)
FUKUI, JAPAN	1972-2012	17	14.5	5.1 (2.3)	5.0 (1.9)	5.0 (1.7)	5.0 (1.5)	5.0 (1.5)	5.0 (1.4)	5.0 (1.3)
FUKUOKA, JAPAN	1972-2012	90	16.8	4.5 (1.7)	4.4 (1.4)	4.4 (1.3)	4.4 (1.2)	4.4 (1.1)	4.4 (1.1)	4.4 (1.0)
GIFU, JAPAN	1972-2012	39	15.7	5.5 (1.9)	5.3 (1.5)	5.3 (1.3)	5.3 (1.2)	5.3 (1.2)	5.3 (1.1)	5.3 (1.1)
GUNMA, JAPAN	1972-2012	38	14.5	5.8 (1.9)	5.6 (1.6)	5.5 (1.4)	5.5 (1.3)	5.5 (1.2)	5.5 (1.2)	5.5 (1.2)
HOKKAIDO, JAPAN	1972-2012	101	8.8	4.7 (2.0)	4.6 (1.7)	4.6 (1.5)	4.7 (1.4)	4.7 (1.3)	4.7 (1.3)	4.7 (1.2)

HIROSHIMA, JAPAN	1972-2012	55	15.9	5.0 (1.8)	4.9 (1.5)	4.8 (1.3)	4.8 (1.2)	4.8 (1.2)	4.8 (1.1)	4.8 (1.1)
HYOGO, JAPAN	1972-2012	98	16.3	4.4 (1.4)	4.3 (1.2)	4.3 (1.1)	4.3 (1.1)	4.3 (1.0)	4.3 (1.0)	4.3 (1.0)
IBARAKI, JAPAN	1972-2012	53	13.6	5.8 (2.5)	5.6 (2.2)	5.6 (2.0)	5.6 (1.9)	5.6 (1.8)	5.6 (1.7)	5.6 (1.7)
ISHIKAWA, JAPAN	1972-2012	23	14.6	4.8 (2.0)	4.7 (1.7)	4.7 (1.6)	4.7 (1.5)	4.7 (1.4)	4.7 (1.3)	4.7 (1.3)
IWATE, JAPAN	1972-2012	30	10.2	5.6 (2.3)	5.5 (2.0)	5.4 (1.8)	5.4 (1.7)	5.4 (1.6)	5.4 (1.5)	5.4 (1.5)
KAGAWA, JAPAN	1972-2012	22	16.1	5.2 (1.9)	5.0 (1.6)	5.0 (1.4)	4.9 (1.3)	4.9 (1.2)	4.9 (1.2)	4.9 (1.2)
KANAGAWA, JAPAN	1972-2012	110	15.7	4.4 (1.5)	4.3 (1.2)	4.3 (1.1)	4.3 (1.1)	4.3 (1.0)	4.3 (1.0)	4.3 (1.0)
KAGOSHIMA, JAPAN	1972-2012	43	18.3	5.0 (2.1)	4.9 (1.9)	4.8 (1.7)	4.8 (1.7)	4.8 (1.6)	4.9 (1.6)	4.9 (1.5)
KOCHI, JAPAN	1972-2012	20	16.9	5.6 (2.1)	5.4 (1.8)	5.3 (1.7)	5.3 (1.6)	5.3 (1.6)	5.3 (1.5)	5.3 (1.5)
KUMAMOTO, JAPAN	1972-2012	39	16.8	5.8 (2.3)	5.6 (1.9)	5.6 (1.7)	5.6 (1.6)	5.6 (1.5)	5.6 (1.5)	5.6 (1.4)
KYOTO, JAPAN	1972-2012	48	15.8	5.5 (2.0)	5.3 (1.6)	5.3 (1.4)	5.3 (1.3)	5.3 (1.2)	5.3 (1.2)	5.3 (1.1)
MIE, JAPAN	1972-2012	36	15.8	4.6 (1.7)	4.5 (1.4)	4.5 (1.2)	4.5 (1.2)	4.5 (1.1)	4.5 (1.1)	4.5 (1.0)
MIYAGI, JAPAN	1972-2012	40	12.4	4.7 (1.8)	4.6 (1.5)	4.6 (1.4)	4.6 (1.3)	4.6 (1.2)	4.6 (1.2)	4.6 (1.2)
MIYAZAKI, JAPAN	1972-2012	24	17.5	5.4 (2.2)	5.2 (2.0)	5.2 (1.9)	5.2 (1.8)	5.2 (1.7)	5.2 (1.7)	5.2 (1.7)
NAGANO, JAPAN	1972-2012	46	11.9	5.9 (2.3)	5.7 (1.9)	5.7 (1.7)	5.7 (1.6)	5.7 (1.6)	5.7 (1.5)	5.7 (1.5)
NAGASAKI, JAPAN	1972-2012	34	17.1	4.3 (1.6)	4.2 (1.4)	4.2 (1.2)	4.2 (1.2)	4.2 (1.1)	4.2 (1.1)	4.3 (1.1)
NARA, JAPAN	1972-2012	24	14.8	6.1 (2.2)	5.9 (1.8)	5.8 (1.6)	5.8 (1.5)	5.8 (1.4)	5.8 (1.3)	5.8 (1.3)
NIIGATA, JAPAN	1972-2012	52	13.8	4.3 (1.9)	4.2 (1.6)	4.2 (1.4)	4.2 (1.3)	4.3 (1.3)	4.3 (1.2)	4.3 (1.2)
OITA, JAPAN	1972-2012	27	16.3	5.1 (2.0)	5.0 (1.7)	4.9 (1.5)	4.9 (1.4)	4.9 (1.3)	4.9 (1.2)	4.9 (1.2)
OKAYAMA, JAPAN	1972-2012	41	15.9	5.4 (1.8)	5.2 (1.5)	5.2 (1.4)	5.1 (1.3)	5.1 (1.3)	5.1 (1.3)	5.1 (1.2)
OKINAWA, JAPAN	1973-2012	18	22.9	3.0 (0.9)	2.9 (0.8)	2.9 (0.7)	2.9 (0.7)	3.0 (0.7)	3.0 (0.7)	3.0 (0.7)
OSAKA, JAPAN	1972-2012	141	16.8	4.7 (1.5)	4.6 (1.3)	4.6 (1.1)	4.6 (1.1)	4.6 (1.0)	4.6 (1.0)	4.6 (0.9)
SAGA, JAPAN	1972-2012	19	16.5	5.4 (2.1)	5.3 (1.7)	5.2 (1.5)	5.2 (1.4)	5.2 (1.3)	5.2 (1.3)	5.2 (1.2)
SAITAMA, JAPAN	1972-2012	89	14.9	5.9 (2.1)	5.7 (1.7)	5.7 (1.5)	5.6 (1.4)	5.6 (1.3)	5.6 (1.3)	5.6 (1.2)
SHIGA, JAPAN	1972-2012	22	14.6	4.7 (1.8)	4.6 (1.5)	4.5 (1.3)	4.5 (1.2)	4.5 (1.1)	4.5 (1.1)	4.5 (1.0)
SHIMANE, JAPAN	1972-2012	19	14.8	5.1 (2.3)	5.0 (1.9)	4.9 (1.7)	4.9 (1.6)	4.9 (1.5)	4.9 (1.4)	4.9 (1.4)
SHIZUOKA, JAPAN	1972-2012	65	16.5	5.1 (2.2)	5.0 (1.8)	4.9 (1.7)	4.9 (1.6)	4.9 (1.5)	4.9 (1.5)	4.9 (1.5)
TOKUSHIMA, JAPAN	1972-2012	19	16.5	4.6 (1.5)	4.5 (1.3)	4.4 (1.2)	4.4 (1.1)	4.4 (1.1)	4.4 (1.0)	4.4 (1.0)
TOCHIGI, JAPAN	1972-2012	37	13.7	6.0 (2.4)	5.8 (2.0)	5.8 (1.9)	5.8 (1.7)	5.8 (1.7)	5.8 (1.6)	5.8 (1.6)

TOKYO, JAPAN	1972-2012	191	16.2	4.4 (1.4)	4.3 (1.2)	4.3 (1.1)	4.3 (1.0)	4.3 (1.0)	4.3 (1.0)	4.3 (0.9)
TOTTORI, JAPAN	1972-2012	14	14.8	5.4 (2.3)	5.3 (2.0)	5.3 (1.8)	5.3 (1.6)	5.3 (1.5)	5.3 (1.4)	5.3 (1.4)
TOYAMA, JAPAN	1972-2012	23	14	5.0 (2.2)	4.9 (1.8)	4.9 (1.7)	4.9 (1.5)	4.9 (1.5)	4.9 (1.4)	4.9 (1.4)
WAKAYAMA, JAPAN	1972-2012	25	16.6	4.8 (1.7)	4.6 (1.4)	4.6 (1.3)	4.6 (1.2)	4.6 (1.1)	4.6 (1.1)	4.6 (1.1)
YAMAGATA, JAPAN	1972-2012	29	11.7	5.6 (2.5)	5.5 (2.2)	5.4 (2.0)	5.4 (1.8)	5.4 (1.8)	5.4 (1.7)	5.4 (1.7)
YAMAGUCHI, JAPAN	1972-2012	36	15.3	5.8 (2.5)	5.7 (2.0)	5.6 (1.8)	5.6 (1.7)	5.6 (1.6)	5.6 (1.5)	5.6 (1.5)
YAMANASHI, JAPAN	1972-2012	18	14.5	6.4 (2.3)	6.2 (1.9)	6.1 (1.7)	6.1 (1.6)	6.1 (1.5)	6.1 (1.5)	6.1 (1.4)
PALMAS G. CANARIA, SPAIN	1990-2010	11	21.3	3.6 (1.0)	3.5 (0.9)	3.4 (0.8)	3.4 (0.8)	3.4 (0.8)	3.4 (0.7)	3.4 (0.7)
TENERIFE, SPAIN	1990-2010	7	21.6	3.7 (1.1)	3.5 (0.9)	3.5 (0.8)	3.5 (0.8)	3.4 (0.8)	3.4 (0.7)	3.4 (0.7)
MELILLA, SPAIN	1990-2010	1	19	4.0 (1.6)	3.9 (1.3)	3.9 (1.2)	3.9 (1.1)	3.9 (1.0)	3.9 (1.0)	3.9 (0.9)
CEUTA, SPAIN	1990-2010	1	18.7	3.5 (1.5)	3.4 (1.3)	3.3 (1.2)	3.3 (1.2)	3.3 (1.2)	3.3 (1.1)	3.3 (1.1)
CÁDIZ, SPAIN	1990-2010	5	18.6	3.7 (1.8)	3.6 (1.6)	3.6 (1.4)	3.6 (1.3)	3.6 (1.2)	3.6 (1.2)	3.6 (1.1)
MÁLAGA, SPAIN	1990-2010	15	18.7	5.6 (2.0)	5.5 (1.6)	5.4 (1.5)	5.4 (1.4)	5.3 (1.3)	5.3 (1.2)	5.3 (1.2)
ALMERÍA, SPAIN	1990-2010	5	19.1	4.9 (1.4)	4.8 (1.2)	4.7 (1.1)	4.7 (1.0)	4.7 (1.0)	4.7 (0.9)	4.6 (0.9)
GRANADA, SPAIN	1990-2010	10	15.7	7.9 (3.2)	7.6 (2.8)	7.5 (2.6)	7.5 (2.5)	7.4 (2.4)	7.4 (2.3)	7.4 (2.3)
HUELVA, SPAIN	1990-2010	6	18.2	6.8 (2.6)	6.6 (2.3)	6.5 (2.1)	6.4 (2.0)	6.4 (1.9)	6.4 (1.8)	6.4 (1.8)
SEVILLA, SPAIN	1990-2010	23	19.5	7.2 (2.8)	6.9 (2.5)	6.8 (2.4)	6.8 (2.3)	6.7 (2.2)	6.7 (2.2)	6.7 (2.1)
JAÉN, SPAIN	1990-2010	5	17	5.3 (2.4)	5.2 (2.2)	5.2 (2.1)	5.2 (2.0)	5.2 (2.0)	5.2 (1.9)	5.2 (1.9)
CÓRDOBA, SPAIN	1990-2010	9	18.3	8.1 (3.8)	7.8 (3.3)	7.7 (3.1)	7.6 (3.0)	7.6 (2.9)	7.5 (2.8)	7.5 (2.8)
MURCIA, SPAIN	1990-2010	10	19	4.0 (1.6)	3.9 (1.3)	3.9 (1.2)	3.9 (1.1)	3.9 (1.0)	3.9 (1.0)	3.9 (0.9)
ALICANTE, SPAIN	1990-2010	7	18.4	5.9 (1.7)	5.7 (1.4)	5.6 (1.3)	5.6 (1.2)	5.5 (1.1)	5.5 (1.1)	5.5 (1.0)
BADAJOZ, SPAIN	1990-2010	5	17.2	8.0 (3.9)	7.6 (3.5)	7.5 (3.3)	7.5 (3.1)	7.4 (3.0)	7.4 (2.9)	7.4 (2.9)
ALBACETE, SPAIN	1990-2010	4	14.4	7.6 (3.5)	7.4 (3.0)	7.3 (2.8)	7.2 (2.6)	7.2 (2.5)	7.2 (2.4)	7.2 (2.3)
CIUDAD REAL, SPAIN	1990-2010	3	15.8	7.3 (3.4)	7.0 (2.9)	6.9 (2.7)	6.9 (2.6)	6.9 (2.5)	6.9 (2.4)	6.9 (2.3)
CÁCERES, SPAIN	1990-2010	5	16.4	6.7 (3.3)	6.4 (3.0)	6.3 (2.8)	6.3 (2.7)	6.3 (2.7)	6.3 (2.7)	6.3 (2.6)
VALENCIA, SPAIN	1990-2010	28	18.5	5.4 (1.9)	5.2 (1.6)	5.1 (1.5)	5.1 (1.4)	5.1 (1.3)	5.1 (1.2)	5.1 (1.1)
PALMA MALLORCA, SPAIN	1990-2010	11	16.7	6.9 (2.2)	6.6 (1.8)	6.5 (1.6)	6.5 (1.5)	6.5 (1.4)	6.4 (1.3)	6.4 (1.3)
TOLEDO, SPAIN	1990-2010	4	15.9	7.5 (3.3)	7.2 (2.9)	7.1 (2.7)	7.1 (2.5)	7.1 (2.4)	7.0 (2.4)	7.0 (2.3)
CASTELLÓN, SPAIN	1990-2010	5	17.8	5.5 (1.5)	5.3 (1.3)	5.3 (1.2)	5.2 (1.1)	5.2 (1.0)	5.2 (1.0)	5.2 (0.9)

CUENCA, SPAIN	1990-2010	2	13.3	7.3 (3.6)	7.1 (3.2)	7.0 (2.9)	7.0 (2.7)	6.9 (2.6)	7.0 (2.5)	7.0 (2.4)
TERUEL, SPAIN	1990-2010	2	12.2	8.7 (3.9)	8.3 (3.4)	8.2 (3.1)	8.2 (2.9)	8.2 (2.8)	8.1 (2.6)	8.1 (2.5)
MADRID, SPAIN	1990-2010	75	15.2	5.8 (2.8)	5.6 (2.5)	5.6 (2.4)	5.6 (2.3)	5.6 (2.3)	5.6 (2.2)	5.6 (2.2)
GUADALAJARA, SPAIN	1990-2010	3	13.3	9.1 (4.5)	8.7 (4.0)	8.6 (3.7)	8.5 (3.5)	8.5 (3.4)	8.5 (3.2)	8.5 (3.2)
AVILA, SPAIN	1990-2010	3	11.2	7.0 (3.6)	6.8 (3.1)	6.7 (2.9)	6.7 (2.8)	6.7 (2.7)	6.7 (2.5)	6.7 (2.5)
SALAMANCA, SPAIN	1990-2010	6	12.3	7.9 (4.2)	7.6 (3.7)	7.5 (3.5)	7.5 (3.3)	7.5 (3.2)	7.4 (3.1)	7.5 (3.0)
SEGOVIA, SPAIN	1990-2010	2	12.4	6.6 (3.2)	6.4 (3.0)	6.3 (2.8)	6.3 (2.7)	6.4 (2.6)	6.4 (2.6)	6.4 (2.5)
TARRAGONA, SPAIN	1990-2010	3	17.9	6.4 (2.2)	6.2 (1.8)	6.1 (1.6)	6.1 (1.4)	6.1 (1.3)	6.1 (1.3)	6.1 (1.2)
BARCELONA, SPAIN	1990-2010	48	16.3	4.9 (1.4)	4.7 (1.2)	4.6 (1.1)	4.6 (1.0)	4.6 (1.0)	4.6 (0.9)	4.6 (0.9)
ZAMORA, SPAIN	1990-2010	3	13.3	7.0 (3.9)	6.8 (3.5)	6.7 (3.3)	6.7 (3.2)	6.7 (3.1)	6.7 (3.0)	6.7 (2.9)
LLEIDA, SPAIN	1990-2010	5	15.2	7.6 (2.8)	7.3 (2.4)	7.2 (2.2)	7.1 (2.1)	7.1 (2.0)	7.1 (1.9)	7.1 (1.9)
VALLADOLID, SPAIN	1990-2010	9	12.9	7.1 (4.0)	6.8 (3.7)	6.8 (3.5)	6.7 (3.3)	6.7 (3.2)	6.7 (3.2)	6.7 (3.1)
ZARAGOZA, SPAIN	1990-2010	19	15.7	6.7 (2.9)	6.5 (2.5)	6.4 (2.3)	6.4 (2.2)	6.4 (2.1)	6.4 (2.1)	6.4 (2.0)
SORIA, SPAIN	1990-2010	2	11.2	7.3 (4.3)	7.1 (3.7)	7.0 (3.4)	7.0 (3.2)	7.0 (3.0)	7.0 (2.9)	7.0 (2.8)
GIRONA, SPAIN	1990-2010	4	14.8	7.4 (2.4)	7.1 (1.9)	7.0 (1.7)	6.9 (1.6)	6.9 (1.5)	6.9 (1.4)	6.9 (1.4)
HUESCA, SPAIN	1990-2010	2	14.1	6.7 (3.1)	6.5 (2.8)	6.4 (2.6)	6.4 (2.6)	6.4 (2.5)	6.4 (2.4)	6.4 (2.4)
BURGOS, SPAIN	1990-2010	5	10.9	7.1 (4.4)	6.9 (3.9)	6.8 (3.7)	6.8 (3.5)	6.8 (3.4)	6.8 (3.2)	6.8 (3.1)
OURENSE, SPAIN	1990-2010	5	15.1	7.7 (4.3)	7.5 (3.7)	7.4 (3.4)	7.3 (3.2)	7.3 (3.1)	7.3 (3.0)	7.3 (2.9)
PONTEVEDRA, SPAIN	1990-2010	4	14.8	5.3 (2.6)	5.1 (2.3)	5.1 (2.1)	5.1 (2.0)	5.1 (2.0)	5.1 (1.9)	5.1 (1.9)
LOGROÑO, SPAIN	1990-2010	4	14	6.9 (3.6)	6.7 (3.1)	6.7 (2.8)	6.6 (2.7)	6.6 (2.6)	6.6 (2.4)	6.7 (2.4)
LEÓN, SPAIN	1990-2010	6	11.1	6.7 (3.6)	6.5 (3.2)	6.4 (3.0)	6.4 (3.0)	6.4 (2.8)	6.4 (2.8)	6.4 (2.7)
PAMPLONA, SPAIN	1990-2010	7	13.1	6.6 (3.7)	6.4 (3.2)	6.4 (2.9)	6.3 (2.8)	6.4 (2.6)	6.4 (2.5)	6.4 (2.5)
VITORIA, SPAIN	1990-2010	5	11.8	6.6 (3.7)	6.5 (3.3)	6.4 (3.0)	6.4 (2.9)	6.4 (2.7)	6.5 (2.6)	6.5 (2.5)
LUGO, SPAIN	1990-2010	5	12.1	6.7 (3.7)	6.5 (3.2)	6.5 (2.8)	6.5 (2.6)	6.5 (2.5)	6.5 (2.3)	6.5 (2.2)
BILBAO, SPAIN	1990-2010	11	14.8	5.7 (2.7)	5.6 (2.2)	5.6 (2.0)	5.6 (1.9)	5.6 (1.7)	5.6 (1.6)	5.6 (1.5)
SAN SEBASTIÁN, SPAIN	1990-2010	9	13.7	3.7 (1.8)	3.7 (1.7)	3.7 (1.6)	3.8 (1.5)	3.8 (1.5)	3.9 (1.5)	3.9 (1.4)
OVIEDO, SPAIN	1990-2010	9	13.3	5.0 (1.9)	4.9 (1.6)	4.9 (1.4)	4.9 (1.3)	4.9 (1.2)	4.9 (1.2)	4.9 (1.1)
A CORUÑA, SPAIN	1990-2010	8	15	3.7 (1.4)	3.6 (1.2)	3.6 (1.1)	3.6 (1.0)	3.6 (1.0)	3.6 (0.9)	3.6 (0.9)
SANTANDER, SPAIN	1990-2010	8	14.6	4.8 (2.2)	4.7 (1.8)	4.7 (1.6)	4.7 (1.5)	4.7 (1.4)	4.7 (1.3)	4.7 (1.3)

CAHUL, MOLDOVA	2003-2010	1	11.3	5.5 (2.9)	5.4 (2.5)	5.4 (2.2)	5.5 (2.0)	5.5 (1.9)	5.6 (1.8)	5.6 (1.7)
CHISINAU, MOLDOVA	2001-2010	15	10.8	5.2 (2.7)	5.1 (2.3)	5.1 (2.1)	5.2 (1.9)	5.2 (1.8)	5.3 (1.7)	5.3 (1.7)
ANENII NOI, MOLDOVA	2003-2010	0	10.5	6.5 (4.0)	6.4 (3.4)	6.4 (3.0)	6.5 (2.8)	6.5 (2.6)	6.5 (2.5)	6.6 (2.4)
FALESTI, MOLDOVA	2003-2010	1	10.2	6.4 (3.9)	6.3 (3.4)	6.3 (3.0)	6.3 (2.7)	6.4 (2.6)	6.4 (2.5)	6.4 (2.4)
EAST, UK	1990-2012	140	10.4	4.7 (2.2)	4.6 (1.9)	4.6 (1.8)	4.6 (1.7)	4.6 (1.6)	4.6 (1.6)	4.6 (1.5)
EAST MIDLANDS, UK	1990-2012	113	10	4.5 (2.0)	4.4 (1.7)	4.4 (1.6)	4.4 (1.5)	4.4 (1.5)	4.4 (1.4)	4.4 (1.4)
LONDON, UK	1990-2012	154	11.6	4.6 (2.1)	4.5 (1.8)	4.5 (1.7)	4.5 (1.6)	4.5 (1.5)	4.5 (1.5)	4.6 (1.5)
NORTH EAST, UK	1990-2012	76	9.5	4.2 (1.9)	4.1 (1.6)	4.1 (1.4)	4.1 (1.3)	4.1 (1.3)	4.2 (1.2)	4.2 (1.2)
NORTH WEST, UK	1990-2012	198	10	4.0 (1.5)	3.9 (1.3)	3.9 (1.2)	3.9 (1.1)	3.9 (1.1)	3.9 (1.1)	3.9 (1.0)
SOUTH EAST, UK	1990-2012	211	10.7	4.5 (2.1)	4.4 (1.8)	4.4 (1.6)	4.4 (1.5)	4.4 (1.5)	4.4 (1.4)	4.4 (1.4)
SOUTH WEST, UK	1990-2012	144	10.6	4.2 (1.7)	4.1 (1.5)	4.0 (1.4)	4.0 (1.3)	4.0 (1.2)	4.1 (1.2)	4.1 (1.1)
WALES, UK	1990-2012	88	10.2	4.2 (1.7)	4.0 (1.4)	4.0 (1.3)	4.0 (1.3)	4.0 (1.2)	4.0 (1.2)	4.1 (1.1)
WEST MIDLANDS, UK	1990-2012	143	10	4.6 (2.0)	4.5 (1.7)	4.5 (1.6)	4.5 (1.5)	4.5 (1.4)	4.5 (1.4)	4.5 (1.3)
YORKSHIRE & HUMBER, UK	1990-2012	140	9.9	4.3 (1.9)	4.2 (1.6)	4.2 (1.5)	4.2 (1.4)	4.2 (1.4)	4.2 (1.3)	4.3 (1.3)
AKRON, OH, USA	1985-2006	13	10.1	6.4 (2.6)	6.4 (2.2)	6.4 (2.0)	6.5 (1.8)	6.5 (1.7)	6.6 (1.7)	6.6 (1.6)
ALBUQUERQUE, NM, USA	1985-2006	9	14.2	8.3 (2.3)	8.0 (2.0)	7.9 (1.8)	7.9 (1.7)	7.8 (1.7)	7.8 (1.6)	7.8 (1.6)
ALLENTOWN-BETHLEHEM, PA, USA	1985-2006	8	11	6.8 (2.8)	6.7 (2.4)	6.7 (2.1)	6.8 (1.9)	6.8 (1.8)	6.8 (1.8)	6.8 (1.7)
ATLANTA, GA, USA	1985-2006	39	17.2	6.4 (2.2)	6.3 (1.9)	6.3 (1.8)	6.3 (1.8)	6.3 (1.7)	6.4 (1.7)	6.4 (1.7)
ATLANTIC CITY, NJ, USA	1985-2006	6	12.2	7.1 (2.6)	7.0 (2.2)	7.0 (2.0)	7.0 (1.9)	7.0 (1.8)	7.1 (1.8)	7.1 (1.7)
AUSTIN, TX, USA	1985-2006	9	20.8	6.9 (2.4)	6.8 (2.1)	6.8 (1.9)	6.8 (1.8)	6.8 (1.8)	6.8 (1.7)	6.8 (1.7)
BAKERSFIELD, CA, USA	1985-2006	11	18.3	8.3 (2.8)	8.0 (2.5)	7.8 (2.3)	7.8 (2.2)	7.8 (2.1)	7.8 (2.1)	7.8 (2.0)
BALTIMORE, MD, USA	1985-2006	40	13.2	6.8 (2.7)	6.7 (2.3)	6.7 (2.0)	6.8 (1.9)	6.8 (1.8)	6.8 (1.7)	6.8 (1.7)
BARNSTABLE-YARMOUTH, MA, USA	1985-2006	6	10.2	5.3 (2.2)	5.2 (1.9)	5.3 (1.7)	5.3 (1.6)	5.4 (1.5)	5.4 (1.5)	5.4 (1.4)
BERGEN-PASSAIC, NJ, USA	1985-2006	30	13.1	5.8 (2.3)	5.8 (2.0)	5.8 (1.8)	5.8 (1.6)	5.9 (1.5)	5.9 (1.5)	6.0 (1.4)
BIRMINGHAM, AL, USA	1985-2006	21	17.2	7.3 (2.8)	7.2 (2.5)	7.2 (2.3)	7.2 (2.2)	7.2 (2.1)	7.2 (2.1)	7.3 (2.0)
BOSTON, MA, USA	1985-2006	59	10.9	5.4 (2.3)	5.4 (1.9)	5.5 (1.8)	5.5 (1.7)	5.6 (1.6)	5.6 (1.5)	5.7 (1.5)
BATON ROUGE, LA, USA	1985-2006	8	20	7.0 (2.5)	6.8 (2.4)	6.8 (2.3)	6.8 (2.2)	6.8 (2.2)	6.8 (2.2)	6.9 (2.2)
BROWNSVILLE, TX, USA	1985-2006	5	23.6	6.1 (2.1)	6.0 (1.9)	6.0 (1.9)	6.0 (1.8)	6.0 (1.8)	6.0 (1.8)	6.0 (1.8)
BUFFALO, NY, USA	1985-2006	26	9.3	5.7 (2.7)	5.8 (2.3)	5.8 (2.0)	5.9 (1.8)	6.0 (1.7)	6.0 (1.6)	6.1 (1.6)

CANTON-MASSILLON, OH, USA	1985-2006	10	10.1	6.4 (2.6)	6.4 (2.2)	6.4 (2.0)	6.5 (1.8)	6.5 (1.7)	6.6 (1.7)	6.6 (1.6)
CHARLESTON, WV, USA	1985-2006	6	13.2	7.2 (3.1)	7.2 (2.6)	7.2 (2.4)	7.2 (2.3)	7.3 (2.2)	7.3 (2.1)	7.3 (2.1)
CHARLOTTE, NC, USA	1985-2006	10	16.2	7.0 (2.5)	6.9 (2.2)	6.9 (2.0)	6.9 (2.0)	6.9 (1.9)	6.9 (1.9)	6.9 (1.9)
CHATTANOOGA, TN, USA	1985-2006	7	16.1	7.2 (2.9)	7.0 (2.5)	7.0 (2.3)	7.0 (2.1)	7.0 (2.1)	7.1 (2.0)	7.1 (1.9)
CHICAGO, IL, USA	1985-2006	139	10	6.4 (2.8)	6.4 (2.4)	6.4 (2.2)	6.5 (2.0)	6.6 (1.9)	6.6 (1.8)	6.7 (1.8)
CINCINNATI, OH, USA	1985-2006	21	12.9	7.0 (2.8)	6.9 (2.4)	6.9 (2.1)	6.9 (2.0)	7.0 (1.9)	7.0 (1.9)	7.1 (1.8)
CLEVELAND, OH, USA	1985-2006	50	10.5	6.1 (2.7)	6.1 (2.3)	6.2 (2.0)	6.3 (1.9)	6.3 (1.8)	6.4 (1.7)	6.4 (1.7)
COLUMBIA, SC, USA	1985-2006	9	17.8	7.8 (3.0)	7.6 (2.7)	7.6 (2.5)	7.6 (2.4)	7.6 (2.3)	7.6 (2.2)	7.6 (2.2)
COLUMBUS, OH, USA	1985-2006	20	11.8	6.6 (2.6)	6.5 (2.2)	6.6 (2.0)	6.6 (1.9)	6.7 (1.8)	6.7 (1.8)	6.8 (1.7)
DALLAS, TX, USA	1985-2006	32	19.1	7.0 (2.3)	6.9 (2.0)	6.9 (1.9)	6.9 (1.8)	6.9 (1.8)	7.0 (1.8)	7.0 (1.7)
DAYTONA BEACH, FL, USA	1985-2006	13	21.8	6.3 (2.3)	6.1 (2.1)	6.0 (2.1)	6.0 (2.1)	6.0 (2.1)	6.0 (2.1)	6.0 (2.1)
DAYTON, OH, USA	1985-2006	14	11.3	6.5 (2.5)	6.4 (2.1)	6.5 (1.9)	6.6 (1.8)	6.6 (1.8)	6.7 (1.7)	6.7 (1.7)
DENVER, CO, USA	1985-2006	23	10.5	9.3 (2.7)	9.0 (2.2)	9.0 (2.0)	9.0 (1.8)	9.0 (1.7)	9.0 (1.6)	9.1 (1.6)
DES MOINES, IA, USA	1985-2006	7	10.4	6.7 (2.6)	6.7 (2.2)	6.7 (2.1)	6.8 (2.0)	6.9 (1.9)	6.9 (1.9)	7.0 (1.9)
DETROIT, MI, USA	1985-2006	91	10.2	6.1 (2.5)	6.0 (2.2)	6.1 (1.9)	6.1 (1.8)	6.2 (1.7)	6.2 (1.6)	6.3 (1.6)
DUTCHESS COUNTY, NY, USA	1985-2006	5	9.8	7.3 (3.0)	7.2 (2.6)	7.2 (2.3)	7.2 (2.1)	7.3 (2.0)	7.3 (1.9)	7.3 (1.8)
EL PASO, TX, USA	1985-2006	9	18.1	8.9 (2.7)	8.6 (2.3)	8.5 (2.1)	8.4 (2.0)	8.4 (1.9)	8.4 (1.9)	8.3 (1.8)
ERIE, PA, USA	1985-2006	7	10.1	5.4 (2.5)	5.5 (2.1)	5.6 (2.0)	5.7 (1.9)	5.8 (1.8)	5.8 (1.7)	5.9 (1.7)
FLINT, MI, USA	1985-2006	9	8.8	6.7 (3.1)	6.7 (2.6)	6.7 (2.3)	6.8 (2.2)	6.8 (2.0)	6.9 (1.9)	6.9 (1.8)
FRESNO, CA, USA	1985-2006	13	18	8.2 (3.6)	7.9 (3.3)	7.7 (3.1)	7.7 (3.0)	7.7 (2.9)	7.7 (2.8)	7.7 (2.8)
FT. LAUDERDALE, FL, USA	1985-2006	38	23	6.0 (2.2)	5.8 (2.0)	5.8 (1.9)	5.7 (1.9)	5.7 (1.9)	5.7 (1.9)	5.7 (1.9)
FORT MYERS-CAPE CORAL, FL, USA	1985-2006	11	24.1	6.3 (1.8)	6.0 (1.6)	6.0 (1.6)	5.9 (1.5)	5.9 (1.5)	5.9 (1.5)	5.9 (1.5)
FORT PIERCE-PORT ST. LUCIE, FL, USA	1985-2006	8	23	6.6 (2.4)	6.3 (2.1)	6.2 (2.0)	6.2 (2.0)	6.2 (1.9)	6.2 (1.9)	6.2 (1.8)
FORT WORTH-ARLINGTON, TX, USA	1985-2006	22	19.1	7.3 (2.4)	7.1 (2.1)	7.1 (2.0)	7.2 (1.9)	7.2 (1.9)	7.2 (1.8)	7.2 (1.8)
GALVESTON, TX, USA	1985-2006	5	20.3	6.2 (2.6)	6.1 (2.4)	6.1 (2.3)	6.1 (2.3)	6.2 (2.2)	6.2 (2.1)	6.2 (2.1)
GARY, IN, USA	1985-2006	11	10	7.1 (3.0)	7.0 (2.5)	7.0 (2.3)	7.1 (2.1)	7.2 (2.1)	7.2 (2.0)	7.2 (1.9)
GRAND RAPIDS, MI, USA	1985-2006	10	9.1	6.3 (2.9)	6.2 (2.5)	6.3 (2.2)	6.3 (2.1)	6.4 (1.9)	6.4 (1.8)	6.5 (1.7)
GREENSBORO, NC, USA	1985-2006	8	14.9	7.0 (2.6)	6.9 (2.2)	6.9 (2.1)	6.9 (2.0)	6.9 (1.9)	6.9 (1.9)	6.9 (1.9)
GREENVILLE, SC, USA	1985-2006	7	16	7.2 (2.7)	7.0 (2.3)	7.0 (2.1)	7.0 (2.0)	7.0 (1.9)	7.0 (1.8)	7.0 (1.8)

HAMILTON, OH, USA	1985-2006	6	12.9	7.0 (2.8)	6.9 (2.4)	6.9 (2.1)	6.9 (2.0)	7.0 (1.9)	7.0 (1.9)	7.1 (1.8)
HARRISBURG-CARLISLE, PA, USA	1985-2006	6	12.1	6.3 (2.7)	6.2 (2.3)	6.2 (2.1)	6.3 (1.9)	6.3 (1.8)	6.3 (1.7)	6.4 (1.7)
HARTFORD, CT, USA	1985-2006	20	10.3	7.0 (2.8)	6.9 (2.4)	6.9 (2.1)	6.9 (2.0)	7.0 (1.8)	7.0 (1.8)	7.0 (1.7)
HONOLULU, HI, USA	1985-2006	13	25.5	4.5 (1.1)	4.3 (1.0)	4.2 (0.9)	4.2 (0.9)	4.2 (0.9)	4.2 (0.8)	4.1 (0.8)
HOUSTON, TX, USA	1985-2006	46	20.9	6.8 (2.5)	6.7 (2.3)	6.7 (2.2)	6.7 (2.1)	6.7 (2.0)	6.7 (2.0)	6.8 (1.9)
INDIANAPOLIS, IN, USA	1985-2006	19	11.8	6.5 (2.4)	6.5 (2.1)	6.5 (1.9)	6.6 (1.8)	6.6 (1.7)	6.7 (1.7)	6.7 (1.7)
JACKSONVILLE, FL, USA	1985-2006	15	20.4	7.1 (2.6)	6.9 (2.4)	6.9 (2.4)	6.9 (2.3)	6.9 (2.3)	6.9 (2.3)	6.9 (2.3)
JERSEY CITY, NJ, USA	1985-2006	13	13.1	5.8 (2.3)	5.8 (2.0)	5.8 (1.8)	5.8 (1.6)	5.9 (1.5)	5.9 (1.5)	6.0 (1.4)
KANSAS CITY, MO-KS, USA	1985-2006	27	12.7	7.0 (2.6)	6.9 (2.2)	6.9 (2.0)	7.0 (1.9)	7.1 (1.9)	7.1 (1.8)	7.2 (1.8)
KNOXVILLE, TN, USA	1985-2006	10	15.1	7.1 (2.8)	7.0 (2.3)	6.9 (2.2)	7.0 (2.1)	7.0 (2.0)	7.0 (2.0)	7.0 (1.9)
LAKELAND-WINTER HAVEN, FL, USA	1985-2006	12	23.1	7.1 (2.0)	6.8 (1.8)	6.7 (1.8)	6.7 (1.7)	6.7 (1.7)	6.7 (1.7)	6.7 (1.7)
LANCASTER, PA, USA	1985-2006	10	11.7	6.8 (2.9)	6.7 (2.4)	6.7 (2.2)	6.7 (2.0)	6.8 (1.9)	6.8 (1.8)	6.8 (1.7)
LANSING, MI, USA	1985-2006	5	8.7	6.9 (3.2)	6.9 (2.7)	6.9 (2.4)	7.0 (2.2)	7.0 (2.1)	7.1 (2.0)	7.1 (1.9)
LAS VEGAS, NV-AZ, USA	1985-2006	23	20.3	7.7 (2.2)	7.4 (1.9)	7.3 (1.7)	7.2 (1.6)	7.2 (1.5)	7.2 (1.5)	7.2 (1.5)
LOS ANGELES, CA, USA	1985-2006	154	17.4	4.7 (2.1)	4.5 (2.0)	4.5 (2.0)	4.5 (2.0)	4.5 (2.0)	4.5 (1.9)	4.5 (1.9)
LOUISVILLE, KY, USA	1985-2006	17	14.4	6.5 (2.5)	6.4 (2.1)	6.5 (1.9)	6.5 (1.9)	6.6 (1.8)	6.6 (1.8)	6.7 (1.8)
LITTLE ROCK, AR, USA	1985-2006	8	17.1	7.1 (2.6)	6.9 (2.2)	6.9 (2.0)	6.9 (1.9)	7.0 (1.9)	7.0 (1.8)	7.0 (1.8)
LUBBOCK, TX, USA	1985-2006	4	16.1	9.0 (2.9)	8.8 (2.6)	8.7 (2.4)	8.7 (2.3)	8.7 (2.3)	8.7 (2.2)	8.7 (2.2)
MADISON, WI, USA	1985-2006	6	8.3	6.8 (3.0)	6.8 (2.4)	6.8 (2.2)	6.9 (2.0)	6.9 (1.9)	7.0 (1.8)	7.0 (1.7)
MCALLEN-EDINBURG-MISSION, TX, USA	1985-2006	6	24.3	6.8 (2.1)	6.7 (1.9)	6.6 (1.8)	6.6 (1.7)	6.7 (1.7)	6.7 (1.7)	6.7 (1.6)
MELBOURNE-TITUSVILLE-PALM BAY, FL, USA	1985-2006	11	22.6	6.1 (2.2)	5.9 (2.1)	5.8 (2.0)	5.8 (1.9)	5.8 (1.9)	5.8 (1.9)	5.8 (1.9)
MEMPHIS, TN, USA	1985-2006	19	17.3	6.5 (2.2)	6.4 (2.0)	6.4 (1.8)	6.4 (1.8)	6.5 (1.8)	6.5 (1.8)	6.6 (1.7)
MIAMI, FL, USA	1985-2006	46	25	4.7 (1.6)	4.5 (1.5)	4.5 (1.4)	4.5 (1.3)	4.5 (1.3)	4.5 (1.3)	4.5 (1.3)
MIDDLESEX, NJ, USA	1985-2006	14	11.7	7.0 (2.7)	6.9 (2.3)	6.9 (2.0)	6.9 (1.9)	6.9 (1.8)	7.0 (1.7)	7.0 (1.6)
MILWAUKEE, WI, USA	1985-2006	29	9.1	5.6 (2.5)	5.6 (2.2)	5.7 (2.0)	5.8 (1.8)	5.9 (1.8)	5.9 (1.7)	6.0 (1.6)
MINNEAPOLIS-ST. PAUL, MN, USA	1985-2006	30	7.9	6.3 (2.6)	6.3 (2.2)	6.3 (2.0)	6.4 (1.9)	6.5 (1.9)	6.5 (1.8)	6.6 (1.8)
MOBILE, AL, USA	1985-2006	9	19.7	6.8 (2.6)	6.6 (2.4)	6.6 (2.3)	6.6 (2.2)	6.6 (2.2)	6.7 (2.2)	6.7 (2.2)
MONMOUTH-OCEAN, NJ, USA	1985-2006	29	11.9	6.3 (2.6)	6.3 (2.3)	6.3 (2.1)	6.3 (2.0)	6.4 (1.9)	6.4 (1.8)	6.4 (1.8)
MYRTLE BEACH, SC, USA	1985-2006	4	17.8	5.7 (2.6)	5.6 (2.4)	5.7 (2.4)	5.7 (2.3)	5.7 (2.3)	5.8 (2.3)	5.8 (2.3)

NAPLES, FL, USA	1985-2006	5	24	6.4 (1.7)	6.2 (1.6)	6.1 (1.5)	6.1 (1.5)	6.0 (1.5)	6.0 (1.4)	6.0 (1.4)
NASHUA, NH, USA	1985-2006	6	8.7	7.4 (3.0)	7.3 (2.5)	7.3 (2.2)	7.3 (2.0)	7.3 (1.9)	7.4 (1.8)	7.4 (1.7)
NASHVILLE, TN, USA	1985-2006	12	15.5	7.0 (2.6)	6.9 (2.3)	6.9 (2.2)	7.0 (2.1)	7.0 (2.0)	7.0 (2.0)	7.1 (1.9)
NASSAU-SUFFOLK, NY, USA	1985-2006	57	11.4	5.7 (2.3)	5.7 (1.9)	5.7 (1.7)	5.7 (1.6)	5.8 (1.5)	5.8 (1.4)	5.8 (1.4)
NEWARK, NJ, USA	1985-2006	28	13.1	5.8 (2.3)	5.8 (2.0)	5.8 (1.8)	5.8 (1.6)	5.9 (1.5)	5.9 (1.5)	6.0 (1.4)
NEWBURGH, NY, USA	1985-2006	6	10.2	7.3 (3.2)	7.2 (2.6)	7.2 (2.4)	7.2 (2.2)	7.2 (2.0)	7.3 (1.9)	7.3 (1.8)
NEW HAVEN-MERIDEN, CT, USA	1985-2006	20	10.3	7.0 (2.8)	6.9 (2.4)	6.9 (2.1)	6.9 (2.0)	7.0 (1.8)	7.0 (1.8)	7.0 (1.7)
NEW LONDON, CT, USA	1985-2006	5	10.7	7.0 (2.9)	6.9 (2.4)	6.9 (2.1)	6.9 (2.0)	6.9 (1.8)	7.0 (1.8)	7.0 (1.7)
NEW YORK, NY, USA	1985-2006	170	13.3	4.9 (1.9)	4.9 (1.7)	5.0 (1.6)	5.0 (1.5)	5.1 (1.5)	5.1 (1.4)	5.2 (1.4)
OAKLAND, CA, USA	1985-2006	40	15.2	4.8 (2.2)	4.6 (2.0)	4.6 (1.8)	4.6 (1.7)	4.6 (1.7)	4.6 (1.6)	4.6 (1.6)
OCALA, FL, USA	1985-2006	7	21.7	7.6 (2.6)	7.3 (2.4)	7.3 (2.3)	7.2 (2.3)	7.2 (2.3)	7.2 (2.2)	7.2 (2.2)
OKLAHOMA CITY, OK, USA	1985-2006	15	16	7.4 (2.6)	7.2 (2.3)	7.2 (2.1)	7.2 (2.0)	7.3 (1.9)	7.3 (1.8)	7.3 (1.8)
OMAHA, NE, USA	1985-2006	9	11	7.4 (3.0)	7.3 (2.5)	7.4 (2.3)	7.4 (2.2)	7.5 (2.1)	7.5 (2.1)	7.5 (2.1)
ORANGE COUNTY, CA, USA	1985-2006	40	18.7	6.8 (2.8)	6.5 (2.5)	6.5 (2.3)	6.4 (2.2)	6.4 (2.1)	6.4 (2.0)	6.4 (1.9)
ORLANDO, FL, USA	1985-2006	20	22.8	6.5 (1.8)	6.3 (1.7)	6.2 (1.7)	6.2 (1.7)	6.2 (1.7)	6.2 (1.7)	6.2 (1.7)
PENSACOLA, FL, USA	1985-2006	6	20.3	6.1 (2.4)	5.9 (2.2)	5.9 (2.1)	5.9 (2.0)	5.9 (2.0)	6.0 (2.0)	6.0 (2.0)
PHILADELPHIA, PA-NJ, USA	1985-2006	113	13.4	6.1 (2.2)	6.0 (1.9)	6.0 (1.7)	6.0 (1.6)	6.1 (1.5)	6.1 (1.5)	6.1 (1.4)
PHOENIX, AZ, USA	1985-2006	48	24	7.7 (2.1)	7.4 (1.8)	7.3 (1.6)	7.2 (1.5)	7.2 (1.4)	7.2 (1.4)	7.2 (1.3)
PITTSBURGH, PA, USA	1985-2006	40	11	6.6 (2.8)	6.6 (2.3)	6.6 (2.1)	6.7 (2.0)	6.7 (1.9)	6.8 (1.8)	6.8 (1.7)
PORTLAND, ME, USA	1985-2006	6	8	6.4 (2.6)	6.4 (2.1)	6.4 (1.9)	6.4 (1.7)	6.4 (1.6)	6.5 (1.5)	6.5 (1.5)
PORTLAND, OR, USA	1985-2006	26	12.5	5.9 (3.6)	5.7 (3.3)	5.7 (3.0)	5.7 (2.9)	5.7 (2.8)	5.7 (2.7)	5.8 (2.7)
PROVIDENCE-FALL RIVER, RI-MA, USA	1985-2006	5	10.9	6.3 (2.3)	6.2 (2.0)	6.2 (1.8)	6.2 (1.6)	6.3 (1.6)	6.3 (1.5)	6.3 (1.4)
PUNTA GORDA, FL, USA	1985-2006	5	23.5	6.8 (2.1)	6.6 (1.9)	6.5 (1.8)	6.4 (1.8)	6.4 (1.8)	6.4 (1.8)	6.4 (1.8)
RALEIGH, NC, USA	1985-2006	7	15.7	7.4 (2.7)	7.3 (2.4)	7.3 (2.2)	7.3 (2.1)	7.3 (2.0)	7.3 (2.0)	7.3 (1.9)
READING, PA, USA	1985-2006	9	15.7	7.4 (2.7)	7.3 (2.4)	7.3 (2.2)	7.3 (2.1)	7.3 (2.0)	7.3 (2.0)	7.3 (1.9)
RIVERSIDE-SAN BERNARDINO, CA, USA	1985-2006	54	19.2	9.3 (3.6)	8.9 (3.1)	8.8 (2.8)	8.7 (2.5)	8.7 (2.4)	8.7 (2.2)	8.6 (2.1)
ROCHESTER, NY, USA	1985-2006	16	9.1	6.3 (2.9)	6.3 (2.4)	6.4 (2.1)	6.4 (2.0)	6.5 (1.9)	6.5 (1.8)	6.6 (1.7)
ROCKFORD, IL, USA	1985-2006	6	9.3	6.8 (2.8)	6.7 (2.4)	6.8 (2.2)	6.8 (2.1)	6.9 (1.9)	6.9 (1.9)	7.0 (1.8)
SACRAMENTO, CA, USA	1985-2006	21	16.4	8.4 (4.3)	8.0 (4.0)	7.9 (3.8)	7.8 (3.7)	7.8 (3.7)	7.8 (3.5)	7.8 (3.5)

SAGINAW, MI, USA	1985-2006	5	8.6	6.0 (2.9)	6.0 (2.5)	6.1 (2.2)	6.1 (2.1)	6.2 (1.9)	6.2 (1.9)	6.3 (1.8)
SALINAS, CA, USA	1985-2006	6	14.4	6.4 (3.2)	6.2 (2.9)	6.2 (2.6)	6.1 (2.5)	6.1 (2.3)	6.1 (2.2)	6.1 (2.2)
SALT LAKE CITY, UT, USA	1985-2006	11	11.7	7.5 (3.1)	7.3 (2.8)	7.2 (2.6)	7.2 (2.5)	7.2 (2.4)	7.2 (2.3)	7.3 (2.3)
SAN ANTONIO, TX, USA	1985-2006	23	20.9	7.2 (2.8)	7.1 (2.5)	7.0 (2.3)	7.1 (2.3)	7.1 (2.2)	7.1 (2.2)	7.1 (2.1)
SARASOTA-BRADENTON, FL, USA	1985-2006	19	23.2	6.4 (2.0)	6.2 (1.8)	6.1 (1.7)	6.1 (1.6)	6.1 (1.6)	6.1 (1.6)	6.1 (1.6)
SCRANTON--WILKES-BARRE--HAZLETON, PA, USA	1985-2006	19	10	6.4 (2.9)	6.4 (2.4)	6.4 (2.2)	6.5 (2.0)	6.5 (1.9)	6.6 (1.8)	6.6 (1.7)
SAN DIEGO, CA, USA	1985-2006	46	17.7	4.1 (2.3)	4.0 (2.2)	3.9 (2.1)	3.9 (2.0)	3.9 (2.0)	3.9 (2.0)	3.9 (2.0)
SEATTLE, WA, USA	1985-2006	28	11.4	5.1 (2.8)	4.9 (2.5)	4.9 (2.3)	4.9 (2.2)	4.9 (2.2)	5.0 (2.1)	5.0 (2.0)
SAN FRANCISCO, CA, USA	1985-2006	31	14.5	5.1 (2.1)	4.9 (2.0)	4.9 (1.8)	4.9 (1.8)	4.9 (1.7)	4.9 (1.6)	4.9 (1.6)
SHREVEPORT, LA, USA	1985-2006	6	18.9	7.2 (2.6)	7.1 (2.3)	7.1 (2.2)	7.1 (2.1)	7.1 (2.0)	7.1 (2.0)	7.1 (2.0)
SAN JOSE, CA, USA	1985-2006	22	16.3	6.8 (2.9)	6.6 (2.5)	6.5 (2.3)	6.4 (2.2)	6.4 (2.1)	6.4 (2.1)	6.4 (2.0)
SPOKANE, WA, USA	1985-2006	9	8.8	6.8 (4.2)	6.6 (3.8)	6.6 (3.6)	6.6 (3.5)	6.6 (3.5)	6.7 (3.4)	6.7 (3.4)
SPRINGFIELD, MA, USA	1985-2006	12	10.3	7.0 (2.8)	6.9 (2.4)	6.9 (2.1)	6.9 (2.0)	7.0 (1.8)	7.0 (1.8)	7.0 (1.7)
STAMFORD-NORWALK, CT, USA	1985-2006	18	11.4	5.4 (2.1)	5.4 (1.8)	5.4 (1.6)	5.4 (1.5)	5.5 (1.4)	5.5 (1.3)	5.5 (1.3)
ST. LOUIS, MO-IL, USA	1985-2006	39	14	6.3 (2.3)	6.3 (1.9)	6.4 (1.8)	6.4 (1.7)	6.5 (1.7)	6.6 (1.6)	6.6 (1.6)
STOCKTON-LODI, CA, USA	1985-2006	10	16.7	8.4 (4.0)	8.1 (3.6)	7.9 (3.4)	7.9 (3.3)	7.8 (3.2)	7.8 (3.1)	7.8 (3.1)
SYRACUSE, NY, USA	1985-2006	11	9.2	6.5 (3.1)	6.5 (2.5)	6.6 (2.3)	6.7 (2.0)	6.7 (1.9)	6.8 (1.8)	6.8 (1.7)
TACOMA, WA, USA	1985-2006	12	11.8	5.4 (2.7)	5.3 (2.4)	5.2 (2.2)	5.2 (2.1)	5.2 (2.0)	5.2 (2.0)	5.2 (1.9)
TAMPA-ST. PETERSBURG-CLEARWATER, FL, USA	1985-2006	20	23	5.9 (1.9)	5.7 (1.7)	5.6 (1.7)	5.6 (1.7)	5.6 (1.7)	5.6 (1.7)	5.6 (1.7)
TOLEDO, OH, USA	1985-2006	11	10.2	6.7 (3.0)	6.7 (2.5)	6.7 (2.3)	6.8 (2.1)	6.8 (2.0)	6.9 (1.9)	6.9 (1.8)
TRENTON, NJ, USA	1985-2006	7	11.7	7.2 (2.7)	7.1 (2.3)	7.1 (2.1)	7.1 (1.9)	7.1 (1.8)	7.1 (1.7)	7.2 (1.6)
TUCSON, AZ, USA	1985-2006	16	21.1	9.3 (2.6)	8.9 (2.3)	8.7 (2.1)	8.7 (2.0)	8.6 (1.9)	8.6 (1.8)	8.6 (1.8)
TULSA, OK, USA	1985-2006	12	16.1	7.1 (2.6)	7.0 (2.3)	7.0 (2.1)	7.0 (2.0)	7.1 (1.9)	7.1 (1.9)	7.2 (1.9)
UTICA-ROME, NY, USA	1985-2006	7	8.2	6.4 (3.0)	6.4 (2.5)	6.4 (2.2)	6.5 (2.0)	6.6 (1.9)	6.6 (1.8)	6.7 (1.7)
VENTURA COUNTY, CA, USA	1985-2006	11	16.2	5.7 (2.5)	5.5 (2.3)	5.4 (2.2)	5.4 (2.1)	5.4 (2.0)	5.4 (2.0)	5.4 (1.9)
VIRGINIA BEACH, VA, USA	1985-2006	23	16	5.9 (2.8)	5.9 (2.4)	5.9 (2.2)	6.0 (2.1)	6.0 (2.1)	6.0 (2.0)	6.1 (2.0)
WASHINGTON, DC-MD-VA, USA	1985-2006	18	14.6	5.9 (2.2)	5.9 (1.9)	5.9 (1.8)	5.9 (1.7)	6.0 (1.6)	6.0 (1.6)	6.0 (1.5)
WICHITA, KS, USA	1985-2006	9	14	7.5 (2.8)	7.4 (2.3)	7.4 (2.2)	7.4 (2.1)	7.4 (2.0)	7.5 (1.9)	7.5 (1.9)
WILMINGTON, DE, USA	1985-2006	9	12.7	6.3 (2.4)	6.3 (2.1)	6.3 (1.9)	6.3 (1.7)	6.3 (1.7)	6.4 (1.6)	6.4 (1.5)

WORCESTER, MA, USA	1985-2006	17	8.8	5.9 (2.2)	5.8 (1.9)	5.9 (1.8)	6.0 (1.7)	6.0 (1.6)	6.1 (1.6)	6.1 (1.5)
WEST PALM BEACH-BOCA RATON, FL, USA	1985-2006	29	24.3	5.1 (2.0)	4.9 (1.8)	4.9 (1.7)	4.9 (1.6)	4.9 (1.6)	4.9 (1.6)	4.9 (1.6)
YORK, PA, USA	1985-2006	8	12.2	7.9 (3.2)	7.7 (2.6)	7.7 (2.4)	7.7 (2.2)	7.7 (2.1)	7.7 (2.0)	7.8 (1.9)
YOUNGSTOWN-WARREN, OH, USA	1985-2006	11	9.7	6.7 (3.1)	6.7 (2.6)	6.8 (2.4)	6.8 (2.2)	6.9 (2.1)	7.0 (2.0)	7.0 (1.9)
ABBOTSFORD, CANADA	1986-2011	3	10.7	5.6 (3.3)	5.5 (2.9)	5.4 (2.7)	5.4 (2.6)	5.5 (2.4)	5.5 (2.3)	5.5 (2.3)
CALGARY, CANADA	1986-2011	14	4.5	7.9 (3.0)	7.8 (2.5)	7.8 (2.2)	7.9 (2.0)	7.9 (1.9)	8.0 (1.9)	8.1 (1.8)
EDMONTON, CANADA	1986-2011	16	4.1	6.8 (2.9)	6.7 (2.5)	6.8 (2.3)	6.9 (2.2)	6.9 (2.2)	7.0 (2.1)	7.1 (2.1)
HALIFAX, CANADA	1986-2011	8	6.7	5.9 (2.5)	5.9 (2.1)	5.9 (1.9)	6.0 (1.7)	6.0 (1.6)	6.0 (1.6)	6.1 (1.5)
HAMILTON, CANADA	1986-2011	12	8.1	6.0 (2.8)	6.0 (2.3)	6.1 (2.1)	6.1 (1.9)	6.2 (1.8)	6.2 (1.7)	6.3 (1.6)
KINGSTON, CANADA	1986-2011	4	7.6	5.7 (2.4)	5.7 (2.0)	5.7 (1.8)	5.8 (1.7)	5.8 (1.6)	5.9 (1.6)	5.9 (1.5)
KITCHENER-WATERLOO, CANADA	1986-2011	8	7	6.5 (3.3)	6.5 (2.7)	6.5 (2.4)	6.6 (2.2)	6.6 (2.0)	6.7 (2.0)	6.7 (1.8)
LONDON ONTARIO, CANADA	1986-2011	10	8.2	6.1 (2.9)	6.1 (2.4)	6.1 (2.1)	6.2 (1.9)	6.2 (1.8)	6.3 (1.7)	6.3 (1.7)
MONTREAL, CANADA	1986-2009	50	6.9	6.0 (2.5)	6.0 (2.2)	6.1 (1.9)	6.2 (1.8)	6.2 (1.7)	6.3 (1.6)	6.3 (1.6)
NIAGARA, CANADA	1986-2011	10	9.3	5.4 (2.3)	5.4 (1.9)	5.4 (1.7)	5.5 (1.6)	5.5 (1.5)	5.6 (1.4)	5.6 (1.4)
OAKVILLE, CANADA	1986-2011	6	8.6	6.0 (2.7)	5.9 (2.3)	5.9 (2.0)	6.0 (1.9)	6.0 (1.7)	6.1 (1.7)	6.1 (1.6)
OSHAWA, CANADA	1986-2011	8	7.6	6.1 (2.6)	6.0 (2.2)	6.1 (1.9)	6.1 (1.8)	6.2 (1.7)	6.2 (1.6)	6.2 (1.5)
OTTAWA, CANADA	1986-2011	14	6.6	6.2 (2.6)	6.2 (2.2)	6.3 (2.0)	6.4 (1.8)	6.4 (1.7)	6.5 (1.6)	6.5 (1.6)
REGINA, CANADA	1986-2011	5	3.1	7.9 (3.5)	7.8 (3.0)	7.8 (2.7)	7.9 (2.6)	8.0 (2.4)	8.0 (2.3)	8.1 (2.2)
SARNIA, CANADA	1986-2011	3	8.8	6.0 (3.0)	6.0 (2.5)	6.1 (2.2)	6.1 (2.1)	6.2 (2.0)	6.2 (1.9)	6.3 (1.8)
SUDBURY, CANADA	1986-2011	4	4.3	6.4 (2.6)	6.4 (2.2)	6.5 (2.0)	6.5 (1.9)	6.6 (1.8)	6.7 (1.8)	6.7 (1.7)
SAINT JOHN NB, CANADA	1986-2011	5	5.3	6.6 (3.1)	6.5 (2.5)	6.6 (2.2)	6.6 (2.0)	6.6 (1.9)	6.7 (1.8)	6.7 (1.8)
ST. JOHN'S NFL, CANADA	1986-2011	6	5.2	5.2 (2.8)	5.2 (2.4)	5.3 (2.1)	5.3 (2.0)	5.4 (1.8)	5.4 (1.7)	5.4 (1.7)
SAULT STE. MARIE, CANADA	1986-2011	3	5.1	6.5 (3.3)	6.4 (2.7)	6.5 (2.4)	6.5 (2.3)	6.6 (2.1)	6.6 (2.0)	6.7 (1.9)
SASKATOON, CANADA	1986-2011	6	2.6	7.7 (3.2)	7.6 (2.8)	7.6 (2.6)	7.7 (2.4)	7.7 (2.3)	7.8 (2.2)	7.9 (2.2)
THUNDER BAY, CANADA	1986-2011	4	3.1	7.8 (3.4)	7.7 (2.8)	7.7 (2.4)	7.7 (2.2)	7.8 (2.1)	7.8 (2.0)	7.8 (1.9)
TORONTO, CANADA	1986-2011	72	8.5	6.0 (2.6)	6.0 (2.2)	6.0 (2.0)	6.1 (1.9)	6.1 (1.8)	6.2 (1.7)	6.2 (1.6)
VICTORIA, CANADA	1986-2011	9	10.2	5.2 (2.5)	5.0 (2.2)	5.0 (2.0)	5.0 (2.0)	5.0 (1.9)	5.0 (1.9)	5.0 (1.8)
VANCOUVER, CANADA	1986-2011	35	10.5	4.3 (1.8)	4.2 (1.6)	4.2 (1.4)	4.2 (1.3)	4.2 (1.3)	4.2 (1.2)	4.2 (1.1)
WINDSOR, CANADA	1986-2011	7	10.1	5.7 (2.5)	5.7 (2.1)	5.7 (1.9)	5.8 (1.8)	5.9 (1.7)	5.9 (1.6)	5.9 (1.5)

WINNIPEG, CANADA	1986-2011	18	3.2	7.2 (3.1)	7.1 (2.6)	7.2 (2.4)	7.3 (2.2)	7.3 (2.1)	7.4 (2.1)	7.5 (2.0)

Table S2: Percent change (mean and 95% CI) of mortality associated with an IQR (inter-quartile range) increase in temperature variability (°C) on different exposure days in cold (four coldest months), hot (four hottest months) and moderate (except four coldest and four hottest seasons) seasons, after controlling for main effect of temperature.

Country	Exposure Days	Percent increase in mortality (%)		
		Cold season	Hot season	Moderate season
Australia	0-1	-0.02 (-0.84, 0.80)	0.37 (-0.03, 0.78)	0.01 (-0.37, 0.40)
Australia	0-2	0.19 (-0.66, 1.04)	0.31 (-0.12, 0.74)	0.10 (-0.57, 0.77)
Australia	0-3	0.37 (-0.34, 1.09)	0.50 (0.10, 0.91)	0.33 (-0.16, 0.82)
Australia	0-4	0.58 (-0.29, 1.45)	0.55 (0.13, 0.98)	0.49 (-0.04, 1.02)
Australia	0-5	0.65 (-0.26, 1.56)	0.57 (0.03, 1.11)	0.59 (0.02, 1.17)
Australia	0-6	0.72 (-0.17, 1.61)	0.64 (0.02, 1.26)	0.70 (0.07, 1.34)
Australia	0-7	0.84 (-0.12, 1.82)	0.79 (0.20, 1.39)	0.85 (0.19, 1.51)
Brazil	0-1	0.82 (0.48, 1.17)	0.72 (0.35, 1.09)	0.78 (0.44, 1.11)
Brazil	0-2	0.61 (0.18, 1.04)	0.54 (0.08, 1.00)	0.59 (0.19, 0.99)
Brazil	0-3	0.48 (0.05, 0.91)	0.44 (-0.04, 0.92)	0.47 (0.06, 0.89)
Brazil	0-4	0.35 (-0.10, 0.81)	0.31 (-0.19, 0.82)	0.37 (-0.08, 0.81)
Brazil	0-5	0.36 (-0.11, 0.83)	0.30 (-0.19, 0.79)	0.38 (-0.08, 0.83)
Brazil	0-6	0.41 (-0.01, 0.84)	0.34 (-0.10, 0.77)	0.46 (0.06, 0.86)
Brazil	0-7	0.47 (0.06, 0.89)	0.39 (-0.07, 0.84)	0.54 (0.14, 0.95)
Thailand	0-1	1.73 (1.19, 2.28)	1.72 (1.23, 2.21)	1.37 (1.05, 1.68)
Thailand	0-2	1.10 (0.56, 1.64)	1.11 (0.64, 1.59)	0.92 (0.65, 1.19)
Thailand	0-3	0.66 (0.10, 1.22)	0.73 (0.22, 1.25)	0.62 (0.31, 0.93)
Thailand	0-4	0.48 (-0.09, 1.05)	0.59 (0.03, 1.15)	0.49 (0.16, 0.82)
Thailand	0-5	0.32 (-0.27, 0.90)	0.42 (-0.17, 1.01)	0.38 (0.03, 0.72)
Thailand	0-6	0.14 (-0.48, 0.75)	0.25 (-0.38, 0.89)	0.27 (-0.08, 0.62)
Thailand	0-7	0.14 (-0.50, 0.78)	0.27 (-0.39, 0.93)	0.26 (-0.09, 0.62)
China	0-1	1.12 (0.36, 1.87)	0.77 (-0.22, 1.78)	0.26 (-0.09, 0.62)
China	0-2	1.21 (0.44, 1.99)	0.80 (0.01, 1.61)	1.36 (0.56, 2.17)
China	0-3	1.17 (0.58, 1.76)	0.87 (0.08, 1.66)	1.31 (0.58, 2.06)
China	0-4	1.43 (0.55, 2.32)	1.10 (0.14, 2.06)	1.65 (0.70, 2.62)
China	0-5	1.21 (0.42, 2.00)	1.04 (0.06, 2.02)	1.58 (0.64, 2.52)
China	0-6	0.92 (0.30, 1.55)	0.98 (0.07, 1.90)	1.46 (0.57, 2.36)
China	0-7	0.86 (0.18, 1.54)	0.93 (0.01, 1.86)	1.45 (0.49, 2.41)
Taiwan	0-1	-0.02 (-0.75, 0.72)	-0.11 (-0.54, 0.33)	0.66 (-0.33, 1.66)
Taiwan	0-2	-0.15 (-0.69, 0.39)	-0.10 (-0.48, 0.29)	0.55 (-0.29, 1.39)
Taiwan	0-3	0.14 (-0.59, 0.88)	0.07 (-0.43, 0.57)	0.75 (-0.30, 1.80)
Taiwan	0-4	0.25 (-0.33, 0.83)	0.14 (-0.28, 0.57)	0.82 (-0.16, 1.81)
Taiwan	0-5	0.17 (-0.39, 0.74)	0.14 (-0.23, 0.50)	0.78 (-0.17, 1.72)
Taiwan	0-6	0.18 (-0.48, 0.85)	0.16 (-0.21, 0.54)	0.81 (-0.19, 1.83)
Taiwan	0-7	0.20 (-0.36, 0.77)	0.20 (-0.14, 0.54)	0.86 (0.13, 1.61)
Korea	0-1	0.31 (0.01, 0.62)	0.28 (-0.11, 0.67)	0.59 (0.08, 1.11)

Korea	0-2	0.39 (0.06, 0.71)	0.34 (0.00, 0.67)	0.56 (0.14, 0.98)
Korea	0-3	0.56 (0.21, 0.91)	0.55 (0.23, 0.88)	0.77 (0.32, 1.21)
Korea	0-4	0.64 (0.33, 0.95)	0.68 (0.35, 1.02)	0.82 (0.47, 1.18)
Korea	0-5	0.74 (0.39, 1.09)	0.80 (0.47, 1.13)	0.85 (0.56, 1.14)
Korea	0-6	0.76 (0.39, 1.14)	0.82 (0.49, 1.16)	0.86 (0.57, 1.16)
Korea	0-7	0.80 (0.47, 1.12)	0.85 (0.52, 1.19)	0.89 (0.57, 1.21)
Japan	0-1	0.15 (0.06, 0.23)	0.26 (0.18, 0.34)	0.56 (0.47, 0.66)
Japan	0-2	0.32 (0.24, 0.39)	0.42 (0.35, 0.50)	0.73 (0.66, 0.80)
Japan	0-3	0.42 (0.35, 0.50)	0.53 (0.46, 0.61)	0.82 (0.75, 0.89)
Japan	0-4	0.56 (0.48, 0.64)	0.63 (0.56, 0.71)	0.93 (0.86, 1.01)
Japan	0-5	0.65 (0.56, 0.73)	0.71 (0.63, 0.79)	1.01 (0.93, 1.08)
Japan	0-6	0.68 (0.60, 0.76)	0.74 (0.67, 0.82)	1.04 (0.96, 1.12)
Japan	0-7	0.72 (0.64, 0.80)	0.78 (0.70, 0.86)	1.08 (0.99, 1.16)
Spain	0-1	-0.02 (-0.27, 0.22)	0.15 (-0.09, 0.40)	0.46 (0.21, 0.72)
Spain	0-2	0.14 (-0.09, 0.38)	0.28 (0.06, 0.50)	0.60 (0.38, 0.82)
Spain	0-3	0.23 (-0.02, 0.49)	0.33 (0.12, 0.55)	0.66 (0.45, 0.88)
Spain	0-4	0.35 (0.09, 0.61)	0.42 (0.21, 0.64)	0.79 (0.55, 1.03)
Spain	0-5	0.41 (0.15, 0.67)	0.48 (0.26, 0.70)	0.85 (0.63, 1.08)
Spain	0-6	0.42 (0.14, 0.70)	0.47 (0.25, 0.70)	0.84 (0.59, 1.08)
Spain	0-7	0.45 (0.16, 0.75)	0.49 (0.26, 0.72)	0.86 (0.60, 1.11)
Moldova	0-1	0.29 (-1.69, 2.30)	0.81 (-0.97, 2.63)	0.28 (-1.52, 2.12)
Moldova	0-2	0.57 (-1.35, 2.54)	1.54 (-0.69, 3.82)	0.93 (-0.87, 2.77)
Moldova	0-3	0.59 (-1.33, 2.56)	1.75 (-1.50, 5.10)	0.95 (-1.04, 2.98)
Moldova	0-4	1.53 (-2.75, 5.99)	2.32 (-2.08, 6.92)	2.20 (-2.71, 7.35)
Moldova	0-5	1.41 (-6.90, 10.46)	1.88 (-3.51, 7.58)	1.37 (-6.67, 10.11)
Moldova	0-6	1.35 (-9.04, 12.93)	1.86 (-3.22, 7.20)	1.61 (-7.51, 11.62)
Moldova	0-7	3.08 (-6.89, 14.11)	2.76 (-2.83, 8.67)	3.10 (-5.84, 12.88)
UK	0-1	0.41 (0.27, 0.55)	0.58 (0.33, 0.83)	0.54 (0.37, 0.71)
UK	0-2	0.31 (0.17, 0.45)	0.44 (0.20, 0.68)	0.43 (0.25, 0.61)
UK	0-3	0.19 (0.03, 0.35)	0.27 (0.03, 0.51)	0.29 (0.08, 0.50)
UK	0-4	0.16 (-0.01, 0.33)	0.21 (-0.04, 0.46)	0.25 (0.02, 0.48)
UK	0-5	0.16 (0.00, 0.32)	0.20 (-0.04, 0.45)	0.24 (0.01, 0.47)
UK	0-6	0.21 (0.04, 0.38)	0.27 (0.01, 0.53)	0.31 (0.07, 0.56)
UK	0-7	0.28 (0.10, 0.46)	0.34 (0.06, 0.62)	0.39 (0.14, 0.65)
USA	0-1	0.33 (0.23, 0.43)	0.37 (0.28, 0.46)	0.69 (0.59, 0.79)
USA	0-2	0.40 (0.29, 0.50)	0.35 (0.25, 0.44)	0.68 (0.59, 0.77)
USA	0-3	0.49 (0.37, 0.60)	0.36 (0.27, 0.46)	0.71 (0.62, 0.81)
USA	0-4	0.57 (0.46, 0.68)	0.38 (0.29, 0.48)	0.75 (0.66, 0.85)
USA	0-5	0.61 (0.50, 0.73)	0.41 (0.31, 0.51)	0.78 (0.68, 0.88)
USA	0-6	0.62 (0.50, 0.75)	0.43 (0.34, 0.53)	0.79 (0.69, 0.89)
USA	0-7	0.67 (0.55, 0.80)	0.47 (0.37, 0.56)	0.82 (0.71, 0.93)
Canada	0-1	0.43 (0.14, 0.72)	0.40 (0.15, 0.66)	0.73 (0.47, 1.00)
Canada	0-2	0.43 (0.12, 0.74)	0.34 (0.12, 0.56)	0.70 (0.44, 0.96)
Canada	0-3	0.50 (0.19, 0.80)	0.31 (0.10, 0.52)	0.70 (0.46, 0.95)
Canada	0-4	0.50 (0.22, 0.77)	0.26 (0.05, 0.47)	0.63 (0.39, 0.87)
Canada	0-5	0.52 (0.26, 0.79)	0.27 (0.04, 0.50)	0.61 (0.36, 0.87)

Canada	0-6	0.55 (0.30, 0.81)	0.28 (0.04, 0.53)	0.62 (0.35, 0.89)
Canada	0-7	0.57 (0.29, 0.85)	0.30 (0.06, 0.54)	0.61 (0.34, 0.88)

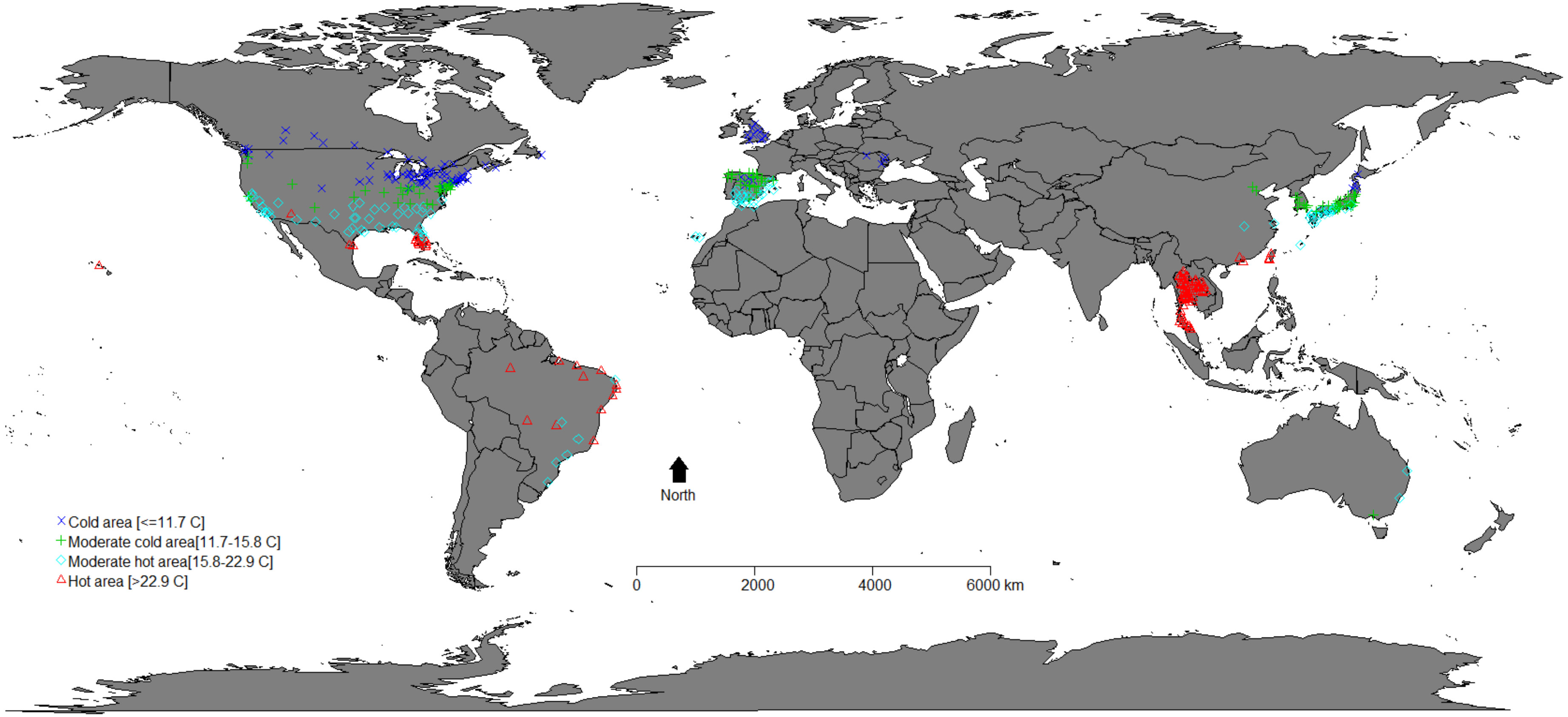

Figure S1: Locations of communities stratified by cold, moderate cold, moderate hot and hot areas, by the quantiles (≤25th, 25th–50th, 50th –75th, and >75th) of their annual mean temperatures (each community has one value of annual mean temperature).

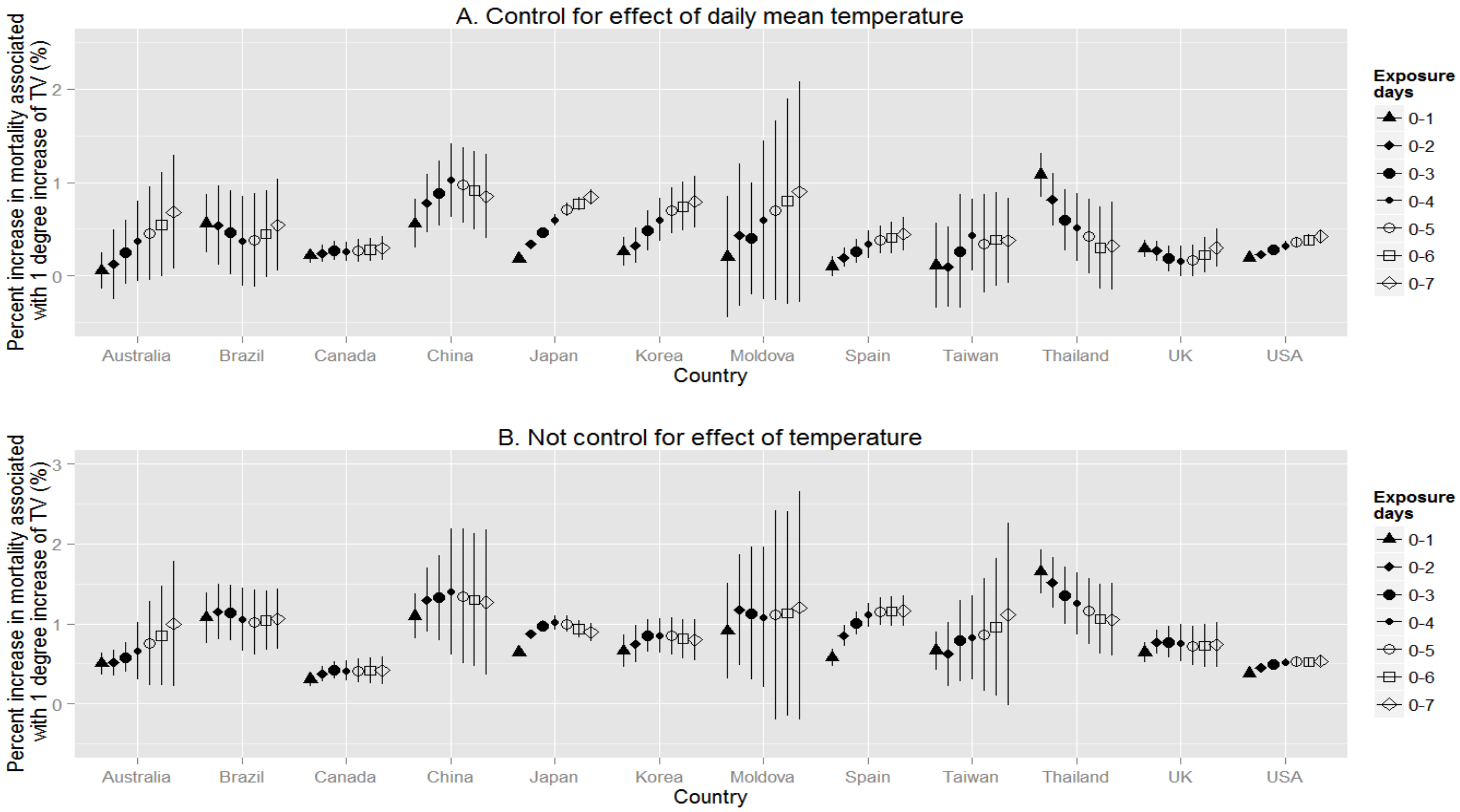

Figure S2: Percent change (95% CI) in mortality associated with one degree increase in temperature variability (°C) on different exposure days, (A) after controlling for the effect of daily mean temperature, (B) without controlling for the effect of temperature.

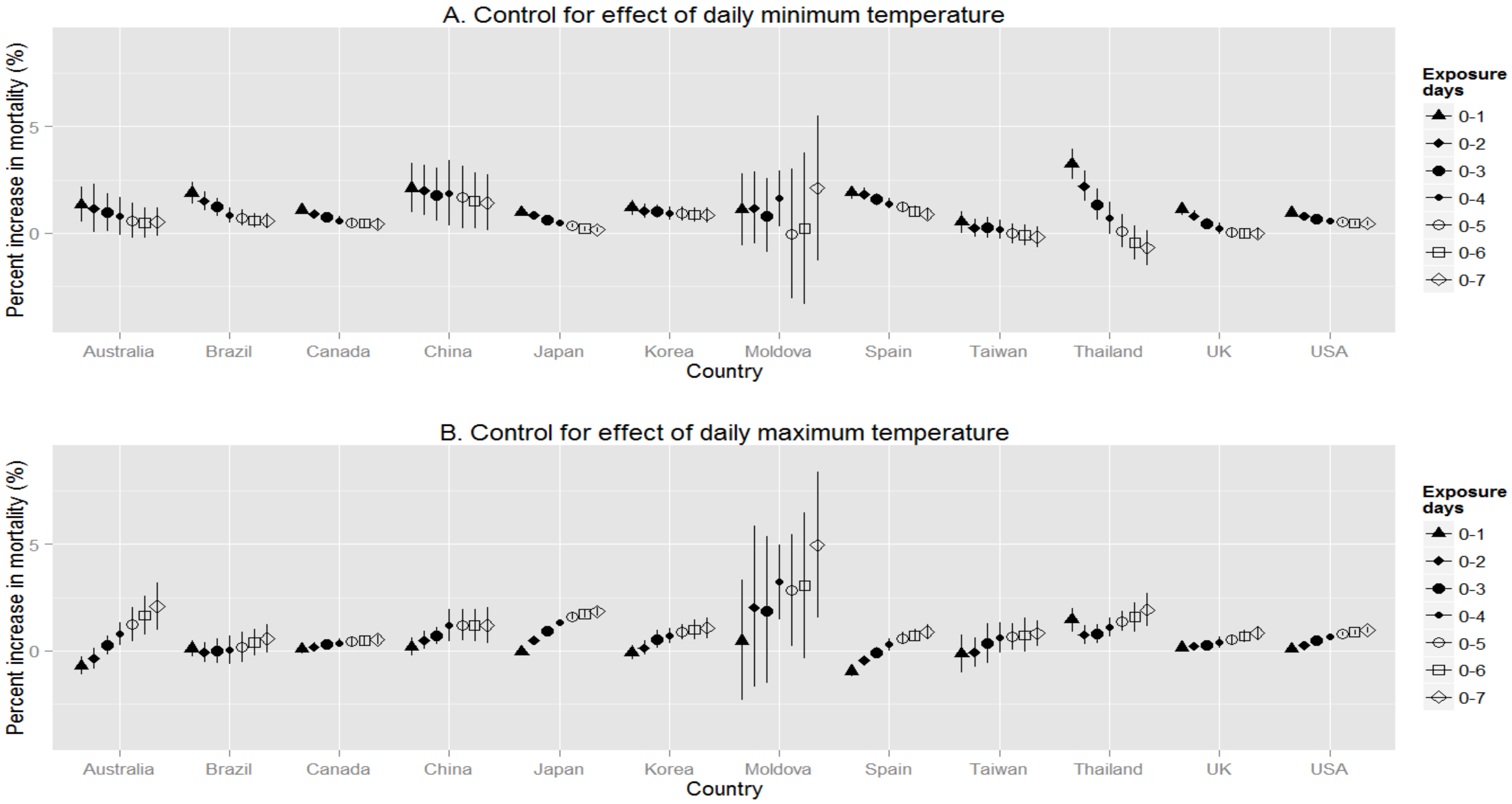

Figure S3: Percent change (95% CI) in mortality associated with an IQR (for each community) increase in temperature variability (ºC) on different exposure days, (A) after controlling for the effect of daily minimum temperature, (B) controlling for the effect of daily maximum temperature.

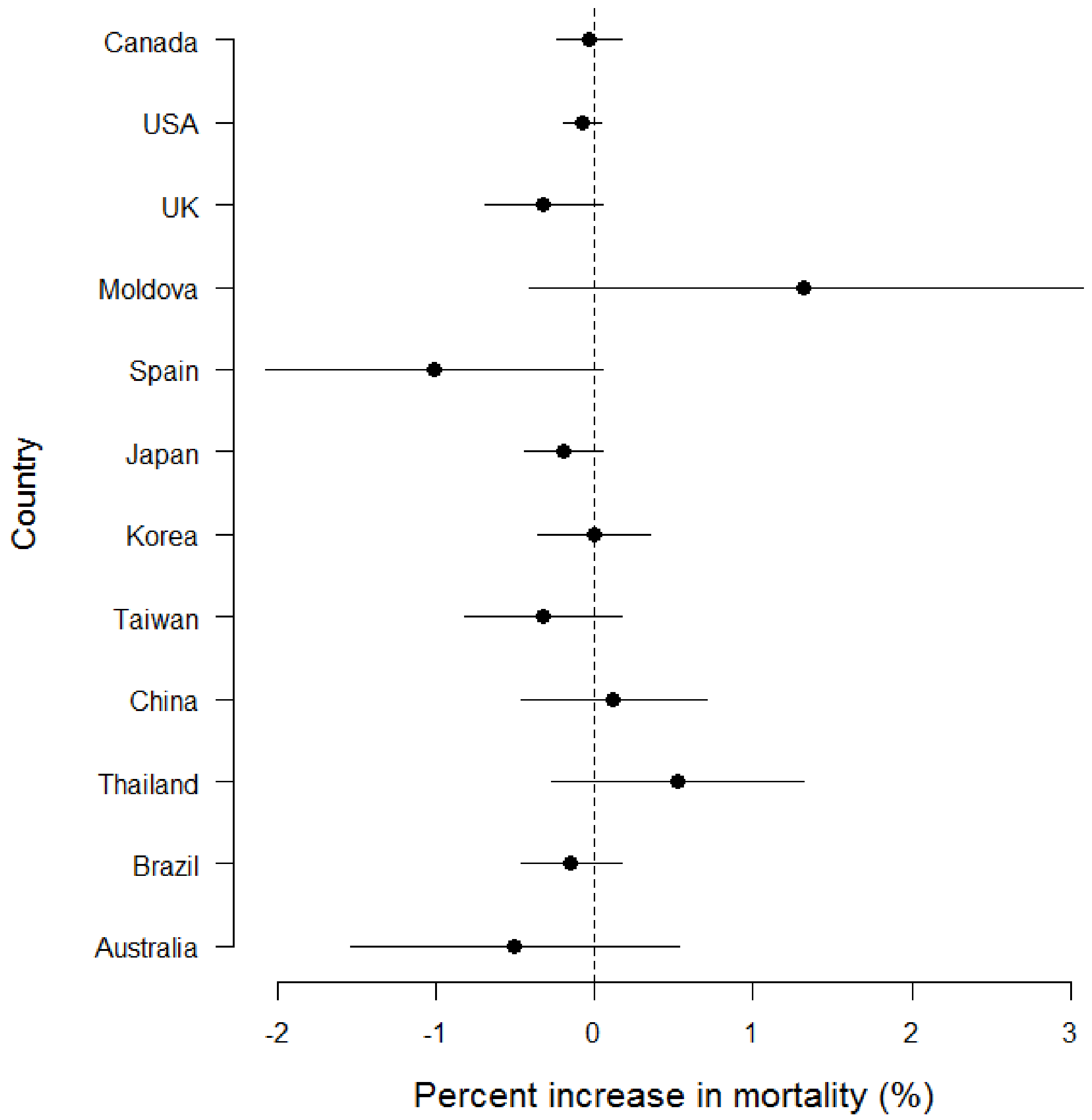

Figure S4: Percent change (95% CI) in mortality associated with future 7 days' temperature variability (°C), after controlling for the main effect of temperature, seasonality, and day of the week.
